# Beyond bloom: validated marker–trait discovery for polyploid roses via GWAS

**DOI:** 10.3389/fpls.2025.1591861

**Published:** 2025-05-13

**Authors:** Laurine Patzer, Dietmar Frank Schulz, Amarachi Queendaline Ezeoke, Marcus Linde, Thomas Debener

**Affiliations:** Institute of Plant Genetics, Department Molecular Plant Breeding, Leibniz University Hannover, Hanover, Germany

**Keywords:** MAS, SNP array, rose ornamental traits, polyploid, molecular markers, genome-wide association studies, QTL, breeding

## Abstract

**Introduction:**

Breeding roses with ideal ornamental characteristics such as beautiful flowers, a pleasant fragrance, and attractive growth habits is complex and time-consuming. This process can be improved and accelerated through the use of molecular markers.

**Methods:**

We conducted a genome-wide association study on nine ornamental traits in roses using the RhWagSNP chip across a panel of 285 cultivars and varieties. Significant marker-trait associations found for major quantitative trait loci were further validated using single-marker analyses with PACE technology in independent panels of up to 182 genotypes.

**Results:**

For six traits— ‘Young shoot: intensity of anthocyanin coloration’, ‘Stem: number of prickles’, ‘Leaf: glossiness of upper side’, ‘Flower: number of petals’, ‘Flower: fragrance’, and ‘Petal: length’—we identified and validated marker-trait associations for major QTLs. Conversely, we were unable to validate associations for ‘Leaf: anthocyanin coloration’ and ‘Leaf: intensity of green color on the upper side’, and found no significant associations in the GWAS for ‘Leaf: size’. Loci that affect petal size, petal number and fragrance have been previously studied. We were able to detect associated markers with increased effect sizes for all three traits. Even greater effects were observed when we combined markers from independent loci for petal number and fragrance.

**Discussion:**

Associated markers for some of the analysed traits largely colocalise with markers previously identified in QTL analyses of biparental populations. Our validation strategy using PACE as an alternative marker technology in independent panels and different environments supports the robustness of our data, irrespective of our limited panel sizes. For the six traits for which we could validate marker-trait associations, our data can be interpreted cautiously as indicating high complexity of inheritance, with few large-effect QTLs influencing the traits. For the other four traits, either greater genetic complexity and/or stronger environmental effects may have confounded our analyses. We believe that the markers presented here can serve as valuable tools for marker-assisted selection and for further genetic analysis of the traits we have analysed.

## 1 Introduction

Roses (Rosa spp.) as members of the Rosaceae family have been valued for centuries as garden, cut and potted plants due to their diverse flower shapes, colors and scents and are among the most important ornamental plants in the world. These aesthetic characteristics determine not only the attractiveness of roses but also their economic value in the ornamental market. Although some breeding objectives differ between cut, potted and garden roses (e.g., vase life vs. hardiness), there are important common criteria for all hybrid roses, such as attractive flower color and flower shape. Depending on the breeding objective, breeding a plant with the desired characteristics can be complex and time-consuming. Flower characteristics such as the number of petals can be selected at the seedling stage, a process applied to approximately 80,000 to 500,000 seedlings per year (Leus et al., 2018; Schulz et al., 2023). More complex flower traits, prickles and growth characteristics are selected only after clonal propagation, and a final decision is made after several years. However, not only is the selection of progeny complex, but the selection of parents also requires the experience of the breeder. To breed individuals with the desired traits, it is usually necessary to cross many parental genotypes. In addition to their phenotypic characteristics, parents must also be selected for fertility and hip production (Pipino et al., 2011, 2013). A Dutch cut rose breeding program consists of an average of 500–600 parent plants that can be selected for crossing (Leus et al., 2018). Knowledge of the genetics of the traits and the use of molecular markers can help reduce the number of crosses and speed up the process of selection in the progeny, thereby reducing costs. For major crops such as barley, maize, tomato, wheat, rice, and soybean, it is common to use marker-assisted selection (MAS), where potential parents and progeny are screened for the presence of alleles that correlate with the desired traits (Xu and Crouch, 2008; Salgotra and Stewart, 2020). In roses, Schulz et al. (2023) showed significant differences between contrasting progeny groups when molecular markers were used to select for rose flower traits. The availability of molecular markers for rose ornamental traits might therefore be of interest to both breeders and the scientific community.

In recent years, significant progress has been made in the field of molecular genetics, especially with the availability of extensive genomic resources and advanced analytical methods. Genome-wide association studies (GWAS) have emerged as powerful methods for identifying the genetic variations underlying complex traits, enabling the identification of statistical associations between genetic markers and phenotypic traits in large, genetically variable populations (Hirschhorn and Daly, 2005; Klein et al., 2005). The statistical models for GWAS are constantly evolving. While GWAS has been used for diploid organisms since the 2000s (Klein et al., 2005), software tools for analyzing polyploid organisms such as roses have been available since 2016 (Rosyara et al., 2016). Roses are characterized by various ploidy levels of the seven chromosomes ranging from diploid to hexaploid, with most cultivated forms being autotetraploid. Some of the chromosomal fragments display segregation pattern between auto- and allotetraploidy (Bourke et al., 2017). Autotetraploids are usually, and in particular in roses, highly heterozygous which can complicate the association of allelic variants with specific traits. Despite these challenges, the development of high-quality rose genome sequences since 2018 (Hibrand Saint-Oyant et al., 2018; Raymond et al., 2018) that represent 96.1% of the estimated genome size of 532.7 Mb, and the WagRhSNP Chip for roses, with 68,893 markers in 2015 (Koning-Boucoiran et al., 2015), have greatly enhanced the precision and efficiency of GWAS in roses. Utilizing these resources, GWAS has been used to establish linked molecular markers for flower traits (Schulz et al., 2016; Hibrand Saint-Oyant et al., 2018; Schulz et al., 2021), callus formation (Nguyen et al., 2020) and adventitious shoot formation (Wamhoff et al., 2023) in roses.

In this study, we used a GWAS to identify new molecular markers for different ornamental traits in a diverse rose gene pool. Based on already published association panels (Schulz et al., 2016; Wamhoff et al., 2023), we increased the population size of our association panels for GWAS analysis up to 285 varieties followed by validation of selected associated markers in independent panels. For some of the traits, we combined markers from different associated loci to increase the predictive power. This knowledge could provide valuable information for future breeding programs and for further investigations on the genetic complexity of the traits studied.

## 2 Materials and methods

### 2.1 Plant material

For the association analysis, 285 varieties and cultivars originating from three panels were used: a panel of 95 garden roses described in Schulz et al. (2016), a set of 95 cut roses described in Wamhoff et al. (2023) and a newly assembled set of 95 garden roses from this study (**Supplementary Table 1**). All garden rose varieties and wild roses were planted in three randomized blocks in a field at Leibniz University Hannover in Herrenhausen (Hanover, Germany). The cut rose genotypes were planted in 7-litre pots with C710 substrate with Cocopor (Stender GmbH, Germany) and cultivated in the greenhouse from mid-December to early May at temperatures of at least 5°C in three replicates.

In addition to the association panels, 190 varieties were used to validate marker-trait associations. Different varieties were used for each trait. These plants were obtained from the collection of the Federal Plant Variety Office (Hanover, Germany) and cultivated in the field in three repetitions.

### 2.2 Phenotyping of the association and validation panels

Rose characteristics were observed according to the relevant CPVO (European Union Community Plant Variety Office) protocol (https://cpvo.europa.eu/sites/default/files/documents/rosa_2_rev.pdf), which is based on documents agreed upon by the International Union for the Protection of New Varieties of Plants (UPOV), such as the General Introduction to DUS (UPOV Document TG/1/3, http://www.upov.int/export/sites/upov/resource/en/tg_1_3.pdf), its associated TGP documents (http://www.upov.int/tgp/en/) and the relevant UPOV Test Guideline TG/11/8 dated 05/04/2006 (https://www.upov.int/edocs/tgdocs/en/tg011.pdf) for the conduct of tests for distinctness, uniformity and stability. Thus, standardized and reproducible assessments across all varieties were ensured.

The following vegetative and floral characteristics were observed:

1. *Young shoot: intensity of anthocyanin coloration (CPVO N°5).* The intensity of anthocyanin coloration on young shoots was visually observed and scored on a scale from very weak (1) to very strong (9).
2. *Stem: number of prickles (CPVO N°6).* The number of prickles on the stems was observed on a defined stem length and internodal position for standardization. It was scored on a nine-point scale from absent or very few (1) to very many (9).
3. *Leaf: size (CPVO N°8).* Leaf size was determined by observing the length and width of mature leaves. It was scored on a nine-point scale from very small (1) to very large (9).
4. *Leaf: intensity of green color (upper side) (CPVO N°9).* The green color intensity of the leaf upper side was visually observed on a nine-point scale from very light (1) to very dark (9).
5. *Leaf: anthocyanin coloration (CPVO N°10).* The presence of anthocyanin coloration on leaf surfaces was visually scored as absent (1) or present (9).
6. *Leaf: glossiness of upper side (CPVO N°11).* Leaf glossiness was visually scored and rated in nine notes ranging from absent or very weak (1) to very strong (9).
7. *Flower: number of petals (CPVO N°22).* The number of petals per flower was counted at full bloom. An average was calculated from at least three flowers per cultivar/variety.
8. *Flower: fragrance (CPVO N°30).* The fragrance of flowers was subjectively evaluated by multiple assessors and categorized in the independent panel as absent or weak (1), medium (2) or strong (3) based on consensus. In the association panel, the original observation scale ranged from weak (1) to strong (7) for the new garden rose panel or from weak (1) to strong (4) for the cut and garden rose panels. This data was then rescaled using the R package ‘caret’ to a standard scale ranging from 0 (weak) to 1 (strong). The rescaling process involved transforming the original scores into a uniform format to ensure that fragrance intensities across different association panels were consistent and comparable.
9. *Petal: length (CPVO N°38).* Petal length was measured in millimeters from the base to the tip of the association panel. In the independent panel, petal length was scored in nine notes from very short (1) to very long (9).

Observations on prickles and leaves were made on the middle third of the stem. Observations of flower characteristics were made only on fully opened flowers at the time of anther dehiscence. Observations of petals were made on a petal from the 3^rd^ outer whorl (in double flowers) or on a petal from the middle whorl (in semidouble flowers).

### 2.3 DNA extraction

For the PACE assay, DNA was extracted from young rose leaves as described by Patzer et al. (2024). DNA for SNP analyses on the WagRhSNP Chip of the new garden rose panel was isolated using the NucleoSpin Plant II Kit from MACHEREY-NAGEL GmbH & Co. KG according to the manufacturer’s instructions. For this purpose, approximately 30–40 mg of leaf tissue was transferred to a reaction vessel and immediately snap frozen in liquid nitrogen. DNA quality and concentration were first assessed using a NanoDrop™ 2000/2000c spectrophotometer (Thermo Fisher Scientific, Inc.) and then diluted to the desired target concentration via intermediate dilution. Final quality and quantity control were conducted using agarose gel electrophoresis with lambda DNA standards. Information on the DNA extraction of the other garden rose panel and the cut rose panel can be found in Schulz et al. (2021); Schulz et al. (2016) and Wamhoff et al. (2023).

### 2.4 Genotyping of the association panel

For the new garden rose panel, SNP analysis was conducted by ATLAS Biolabs GmbH (Berlin, Germany). Genotyping of all three association panels was performed using the WagRhSNP chip, a 68k SNP array designed for roses (Koning-Boucoiran et al., 2015). On the basis of the signal intensities of the samples, allele dosage calling was conducted using a combination of the R packages SNPolisher (Thermo Fisher Scientific Inc, 2020) and FitTetra (Voorrips et al., 2011), both specifically designed to handle the complexities of tetraploid genotyping. After dosage determination, each SNP was analyzed for its genomic position. For this purpose, the SNP data were related to the Rosa chinensis Genome v1.0 (Hibrand Saint-Oyant et al., 2018). The complete dataset (with information on allele dosage calls and positions in the genome) contained 38,863 (new garden rose panel), 37,161 (garden rose panel) and 36,874 (cut rose panel) unique SNPs, including chromosome 0.

### 2.5 GWAS analysis

The associations between SNP markers and phenotypes were analyzed using the R package GWASPoly (Rosyara et al., 2016). The K model with leave-one-chromosome-out (K.loco) was used to control the population structure, which was tested for suitability using QQ plots (data not shown). The threshold value of 1-5/N with N= population size was set for the maximum genotype frequency to exclude markers with little validity. The LD-corrected variant of the Bonferroni threshold (‘M.eff’) was selected as the significance threshold. The GWAS was conducted using both an additive model and a simplex dominance model. Effect sizes (β), representing the estimated change in the trait per allele substitution, were calculated in GWASPoly from the regression coefficients obtained in the association analysis, quantifying the impact of significant SNPs on trait variation. To estimate the proportion of phenotypic variance explained by each significant marker, R² values were calculated using the fit.QTL function in GWASPoly. This function builds a multiple QTL model and estimates the partial R² value for each QTL through backwards elimination.

The GWAS results were visualized with the R package ggplot2 (Wickham, 2016).

### 2.6 Marker selection for verification

Markers were selected from each significant region in the GWAS analysis, ensuring that at least two markers were chosen whenever possible. Subsignificant markers were selected from regions where adjacent markers exhibited an association signal (‘peaks’), that were near the threshold. Alternatively, markers were also considered if they demonstrated significant associations in the GWAS analysis of one panel. Markers based on publications by Schulz et al. (2021) and Hibrand Saint-Oyant et al. (2018) were used as reference markers.

### 2.7 PACE^®^ assay for SNP verification of individual markers

For each marker selected for verification, two allele-specific forward primers and one common reverse primer were designed. The primer design was based on sequences 50 bp around the SNP position and was performed using the PACE^®^ Assay Design Template from 3CR Bioscience Ltd. The specific primer sequences can be found in **Supplementary Table 2**. The PACE^®^ assay was performed using PACE^®^ 2.0 Genotyping Master Mix with a low Rox concentration according to the manufacturer’s instructions. For a 384-well plate, 2.5 µL of DNA (15 ng) and 2.5 µL of master mix, including the assay mix, were combined in each reaction using the liquid handling system epMotion^®^ 5075t from Eppendorf SE. Thermal cycling and fluorescent signal detection were conducted with a QuantStudio™ 6 Flex Real-Time PCR System (Thermo Fisher Scientific, Inc.). When a newly designed assay mix was used, 26 to 39 PCR cycles were tested. Because the QuantStudio software used cannot score tetraploid organisms, the fitPoly package (Voorrips and Gort, 2018) from the statistical software R (R Core Team, 2024) version 4.0.4 was used for SNP dosage calling. Here, dip.filter=0 and p.threshold=0.95 were used instead of the default settings.

### 2.8 Statistical analysis

All the statistical analyses were performed using *R* software, version 4.4.0 (R Core Team, 2024). The distribution of the data was assessed using visualizations such as histograms, facilitated by the `ggplot2` data visualization package (Wickham, 2016). Correlations between variables were evaluated using the Pearson correlation coefficient. The correlation analyses and visualizations were performed using the `corrplot` package (Wei and Simko, 2021). Statistical significance was accepted at a level of α = 0.05. To assess the impact of allele dosage on the traits of interest, the nonparametric Kruskal–Wallis test was employed. The test was conducted using the `stats` package, which is part of the base R distribution. *Post hoc* pairwise comparisons were performed using Dunn´s test with Bonferroni adjustment. The effect size (η²) was also calculated as part of this analysis. η² is derived from the Kruskal–Wallis test and represents the proportion of variance in the traits that can be attributed to the differences among groups, in this case, the different allele dosage levels. For traits with a binary (yes/no) distribution, the Pearson´s Chi-squared test was used to evaluate the association between allele dosage and trait expression.

## 3 Results

### 3.1 Phenotypic analysis of nine rose ornamental traits

**Figure 1:**
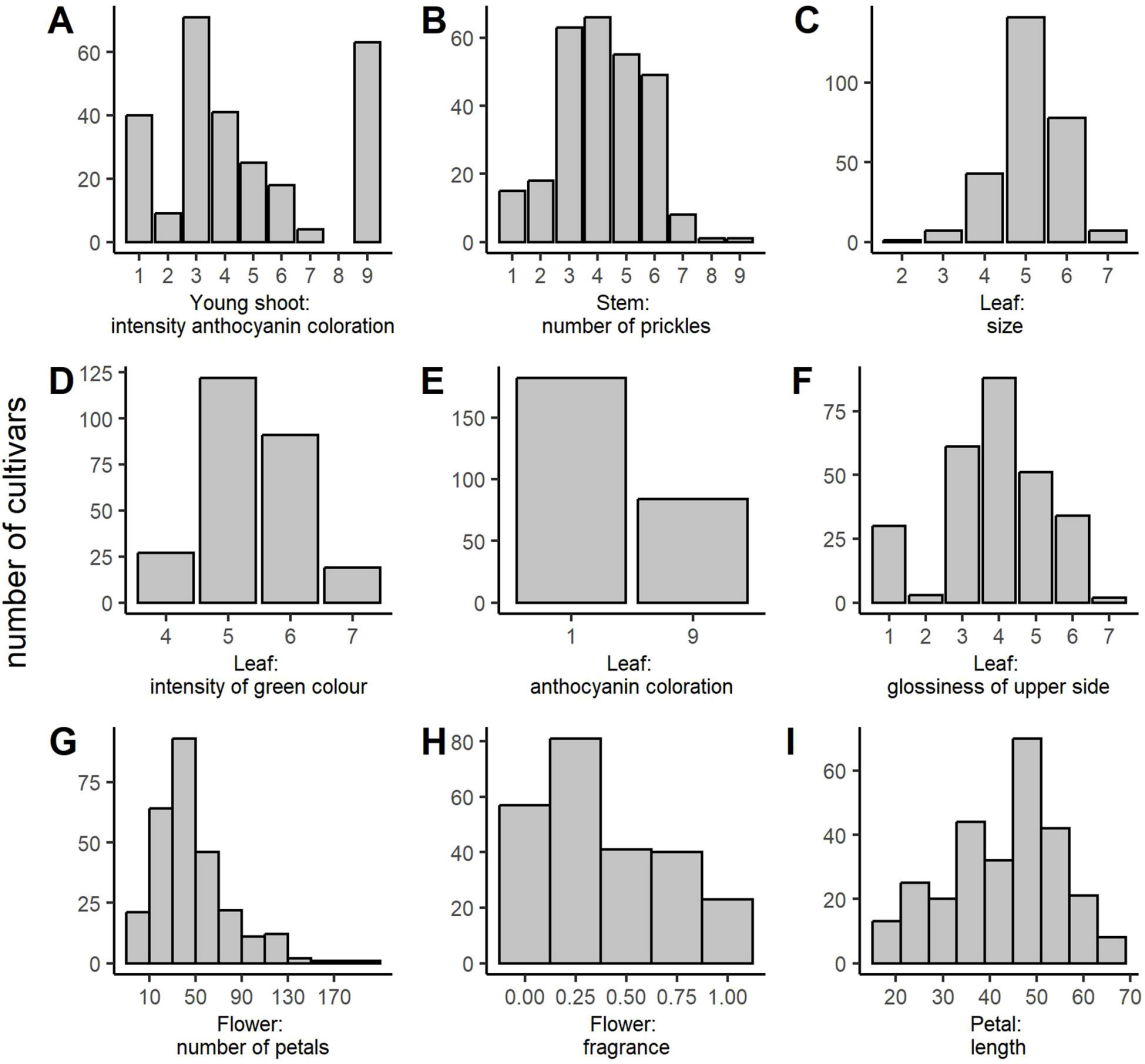
Distribution of the phenotypic classes for all ornamental traits analyzed in the association panel. **(A)** Young shoot: intensity of anthocyanin coloration, **(B)** Stem: number of prickles, **(C)** Leaf: size, **(D)** Leaf: intensity of green color (upper side), **(E)** Leaf: anthocyanin coloration, **(F)** Leaf: glossiness of upper side, **(G)** Flower: number of petals, **(H)** Flower: fragrance, and **(I)** Petal: length (in mm).

Phenotypic data were collected at different scales ranging from metric measurements (number of petals and petal length) to interval-scaled scores (e.g., 0–1 for ‘Flower: fragrance’ or 1–9 for ‘Leaf: anthocyanin coloration’). Analysis of the distribution of the phenotypic data revealed that the majority of the metric and interval-scaled traits approximately followed a normal distribution (**Figure 1**). However, some traits (e.g., ‘Young shoot: anthocyanin coloration’, ‘Flower: number of petals’, and ‘Flower: fragrance’) slightly deviated from the normal distribution, as indicated by asymmetries and outliers in the histograms. Despite this, the phenotypic data were not transformed because 1) the methods used in GWAS analyses are robust to small deviations from the normal distribution, and we verify the markers on an independent population; 2) the effect sizes are easier to interpret with the original data; and 3) transforming the data could bias the dataset and obscure more subtle traits. The distributions in the independent panel were similar for all the traits except for ‘Young shoot: anthocyanin coloration’ and ‘Flower: fragrance’ (**Supplementary Figure 1**).

**Figure 2:**
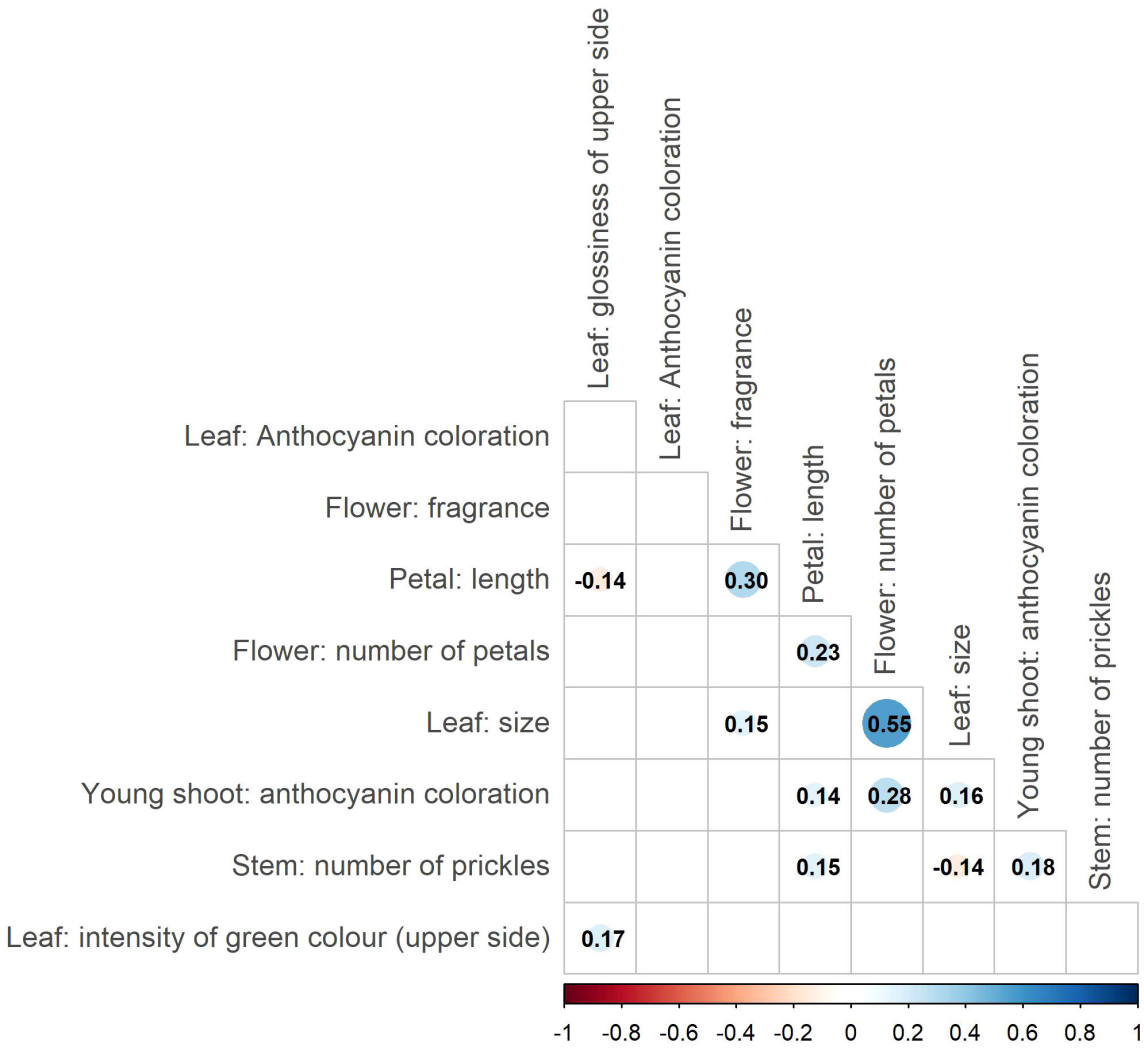
Correlations between ornamental traits based on the association panels. Only significant correlations (p<0.05) are highlighted by color codes, and numbers represent Pearson’s correlation coefficient for the respective combinations. The size of the points indicates the strength of the correlation.

Correlation analysis of the nine phenotypic traits revealed several significant correlations (**Figure 2**). However, most of them were weak (|r| < 0.3), indicating an independent variation/inheritance and thus providing valuable input for the association analyses. Notably, moderate correlations were detected between the traits ‘Petal: length’ and ‘Flower: fragrance’ (r = 0.3, p < 0.05), as well as between ‘Flower: number of petals’ and ‘Leaf: size’ (r = 0.55, p < 0.05).

### 3.2 GWAS analysis of rose ornamental traits

**Figure 3:**
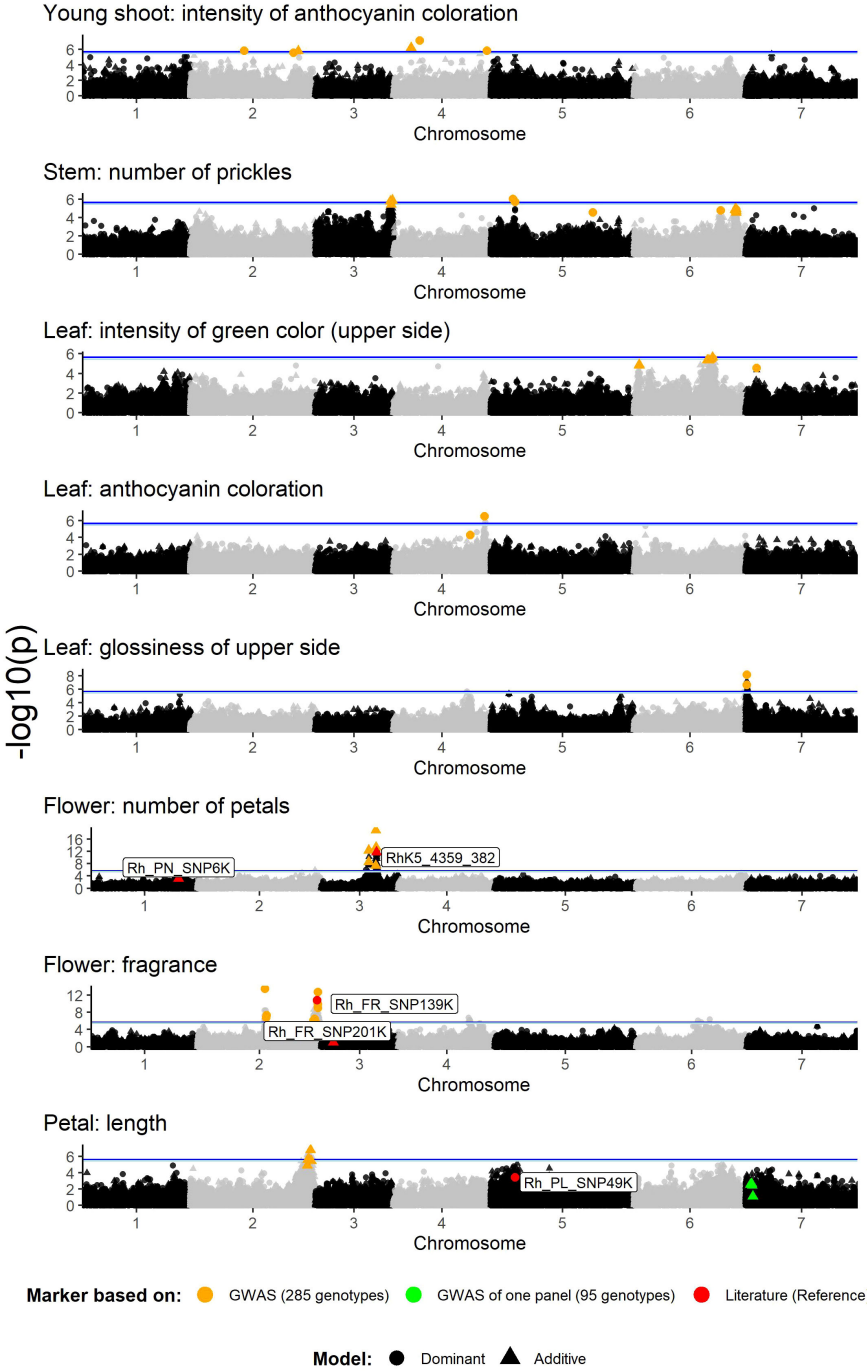
Manhattan plots for the marker–trait associations of rose ornamental traits in a set of 285 garden and cut roses. The selected SNP markers for verification are marked in orange. The significance threshold (M.eff) is indicated as a blue line (light blue for the dominant model with M.eff_dom_= 5.44 and dark blue for the additive model with M.eff_add_= 5.63) and was determined on the basis of the LD-corrected Bonferroni threshold (‘M.eff’). Orange = selected markers on the basis of this GWAS result; green = selected markers significant in only the new garden rose panel; red = reference markers from Schulz et al. (2021) and Hibrand Saint-Oyant et al. (2018). The x-axis indicates the genomic position of the markers, and the y-axis indicates the negative logarithmic p value (-log10 (p)) for each marker–trait association. Each dot represents a single SNP, and the horizontal line indicates the significance threshold.

GWAS analysis of the association panel of 285 genotypes using the 68k WagRhSNP chip revealed several highly significant associations between SNP markers and eight of the nine phenotypic traits analyzed (**Figure 3**). For the trait ‘Leaf: size’, no marker peaks beyond the significance threshold were found (**Supplementary Figure 2**).

#### 3.2.1 New traits analyzed via GWAS for the first time

*Young shoot: anthocyanin coloration*

For this trait, significant marker–trait associations were found on chromosomes 2 and 4, which are located at five different loci (**Figure 3**). In total, seven significant markers with –log (p) values ranging from 5.53 to 7.13 (dominant model) and 5.77 to 6.2 (additive model) were selected (**Table 1**). The effect sizes ranged between |β|=1.556 and 2.785 (dominant model) resp. 1.361 and 2.089 (additive model) on a scale of 1 to 9.

**Table 1:** Selected SNP markers for validation of the GWAS results on marker–trait associations.

Trait	Marker	Chrom	Position [Mb]	–log(p)additive	Effect (β) additive	-log(p) 1-dom	Effect (β) 1-dom	Effect (η^2^) Eta2[H]
Young shoot: intensity of anthocyanin coloration	RhK5_17994_176	2	32.19			5.79	1.556	0.095
	Rh12GR_43585_254	2	61.81			5.55	-2.340	0.14
	Rh12GR_30029_334	2	62.05			5.54	-2.401	0.116
	RhK5_1892_483	2	64.79	5.77	-2.089	5.53	-2.666	n.s.
	Rh12GR_38290_1803	4	10.89	6.2	1.361			0.119
	Rh12GR_19603_664	4	16.03			7.13	-2.785	0.127
	RhMCRND_5216_974	4	56.39			5.81	-2.436	0.143
Stem: number of prickles	RhMCRND_15645_551	3	45.18	5.75	-0.582			0.116
	RhMCRND_33463_124	3	45.60	5.42	n.s.			0.101
	RhK5_12076_566	3	46.34	5.87	-0.575			0.075
	RhK5_1809_431	5	13.29			6.02	-1.450	n.s.
	Rh12GR_16070_248	5	14.39			5.71	1.373	n.s.
	RhMCRND_2230_1005	5	61.17			4.56	n.s.	0.081
	RhK5_432_2321	6	52.30			4.76	n.s.	n.s.
	RhK5_5501_1135	6	60.71	4.85	n.s.			0.063
	RhMCRND_12154_179	6	61.42	4.68	n.s.			0.08
	RhK5_4270_45	6	61.45	4.52	n.s.			n.s.
	RhK5_3587_1335	6	61.47	4.90	n.s.			0.062
	RhMCRND_4282_2838	6	61.61			4.89	n.s.	0.08
Leaf: intensity of green color	RhMCRND_201_1253	6	3.16	4.80	n.s.			0.069
	RhMCRND_32494_229	6	3.26	4.91	n.s.			0.08
	RhK5_8654_792	6	44.12	5.35	n.s.			0.081
	RhK5_16131_601	6	45.11	5.37	n.s.			0.083
	RhK5_2954_1267	6	47.27	5.63	n.s.			0.078
	RhMCRND_18571_180	6	47.45			5.52	0.649	0.069
	Rh88_48595_614	7	6.54			4.55	n.s.	0.105
Leaf: anthocyanin coloration	RhK5_5599_259	4	46.25			4.28	n.s.	NA
	RhMCRND_1644_1712	4	54.98			6.53	-4.104	NA
Leaf: glossiness of upper side	Rh12GR_4274_338	7	0.54	7.15	-0.576	8.17	-1.145	0.194
	RhK5_14250_324	7	0.57			6.65	0.893	0.174
Flower: number of petals	**Rh_PN_SNP6K (Ref)**	1	53.16	3.09	n.s.			
	Rh12GR_54461_324	3	28.84	8.59	-12.196			0.225
	RhMCRND_10097_334	3	28.99	12.35	-14.855			0.278
	RhMCRND_13217_328	3	33.15	7.40	12.133			0.156
	RhMCRND_760_1054	3	33.22	18.91	-18.960	6.55	-37.072	0.348
	RhK5_10101_93	3	33.23	13.39	18.511			0.279
	**RhK5_4359_382 (Ref)**	3	33.56	11.79	-16.807			0.252
Flower: fragrance	RhMCRND_13639_80	2	40.96	6.68	-0.109	13.45	-0.352	0.201
	Rh12GR_62784_393	2	41.52			6.74	0.378	0.093
	Rh12GR_53908_964	2	41.89			7.38	-0.279	0.092
	RhMCRND_6741_1060	2	70.42	5.82	-0.094	6.11	-0.206	0.285
	RhMCRND_4712_444	2	71.06			6.48	-0.201	0.271
	RhMCRND_5437_1194	2	72.29	5.81	0.109			0.264
	RhK5_18439_164	2	72.42	7.35	0.129	10.67	0.311	0.418
	**RhMCRND_11924_839 (Ref)**	2	72.49	6.79	-0.124	10.85	-0.304	0.362
	RhMCRND_2744_848	2	73.01	7.67	-0.111	9.06	-0.298	0.355
	RhK5_12307_104	2	73.03	8.17	0.112	9.91	0.289	0.399
	RhMCRND_12686_297	2	73.09	5.97	-0.122	12.73	-0.358	0.364
	**Rh_FR_SNP201K (Ref)**	3	7.25	1.05	n.s.			
Petal: length	Rh12GR_92884_1039	2	70.19	6.75	-2.703			0.04
	RhMCRND_6741_1060	2	70.42	6.83	-2.715			0.258
	RhK5_14720_826	2	71.15	5.73	-2.552			0.03
	RhK5_10683_422	2	72.26	6.8	2.676			0.014
	RhK5_12478_1400	2	72.72	6.73	-2.918			0.064
	**Rh_PL_SNP49K (Ref)**	5	14.51			3.47	n.s.	
	RhK5_18872_1065	7	2.84	*5.57	*0.441			*0.311
	RhMCRND_982_1009	7	3.57	*6.67	*0.680			*0.363
	RhK5_3530_858	7	3.85	*5.89	*-0.434			*0.373
	RhK5_1987_433	7	4.29	*6.17	*0.443			*0.32

The threshold for the dominant model was 5.44, and that for the additive model was 5.63. *derived from the new garden rose panel, with a threshold of 5.54 (additive model). Chrom, chromosome. Markers in bold were previously described in publications by Hibrand Saint-Oyant et al. (2018) and Schulz et al. (2021). The markers in red are significant for two separate phenotypic traits. ns, not significant.

*Stem: number of prickles*

The analysis of the prickle number revealed two significant peaks on chromosomes 3 (45–46 Mb) and 5 (13–14 Mb), from which five markers were selected with –log (p) values of 5.42 (additive model) to 6.02 for the dominant model (**Figure 3**; **Table 1**). In addition, seven markers were selected from the subsignificant peaks on chromosomes 5 and 6.

*Leaf: intensity of green colourcolor on the upper side*

Here, one significant marker peak was observed on chromosome 6 (47 Mb), with a maximum –log (p) value of 5.63 for the additive model (**Figure 3**). Subsignificant peak regions were observed at 3 Mb on chromosome 6 and at 7 Mb on chromosome 7, from which three markers were selected for testing (**Figure 3**; **Table 1**).

*Leaf: anthocyanin coloration*

For this trait, a clear peak was identified on chromosome 4 (55–56 Mb). From this peak, one significant marker following the dominant model, RhMCRND_1644_1712, with an effect size of β=-4.104 on a scale of 1 to 9, was selected (**Figure 3**; **Table 1**). In addition, a nonsignificant marker from an adjacent region at 46 Mb (RhK5_5599_259) was chosen.

*Leaf: glossiness of upper side*

For this trait, one significant peak was observed on chromosome 7 at 0.5–0.6 Mb. From this peak, two dominant markers, Rh12GR_4274_338 and RhK5_14250_324, were selected (**Figure 3**). Both markers were significant, with –log (p) values of 7.15 and 6.65 (dominant model), respectively, and displayed effect sizes ranging from β=-0.576 to β=0.893 [1, 9] (**Table 1**).

#### 3.2.2 Traits that were reanalyzed via GWAS

The following three traits were previously analyzed by GWAS in a garden rose panel of 95 genotypes (Schulz et al., 2021) and were reanalyzed using statistical software developed for tetraploids in the extended GWAS panel.

*Flower: number of petals*

GWAS analyses of petal numbers revealed two highly significant peaks on chromosome 3, with –log (p) values reaching 12.35 (Peak PN1) and 18.91 (Peak PN2) for the additive model (**Figure 3**; **Table 1**). Peak PN1, located in the region of 28–29 Mb, included two markers (Rh12GR_54461_324 and RhMCRND_10097_334) with a maximum effect size of |β|=14.85. Peak PN2, spanning 33–34 Mb, included four markers (RhMCRND_13217_328, RhMCRND_760_1054, RhK5_10101_93 and RhK5_4359_382) with a maximum effect size of |β|=37.072 [0, 200]. The marker RhK5_4359_382 (β= -16,807) was also found in the studies of Hibrand Saint-Oyant et al. (2018) and was used as a reference marker for the validation panel (see next chapter). Furthermore, a validated marker for petal number from previous studies (Schulz et al., 2021) was located on chromosome 1 in a region where a less significant peak was found (**Figure 3**).

*Flower: fragrance*

Here, two distinct peaks from 40–42 Mb (Peak Fr1) and 70–73 Mb (Peak Fr2) were observed on chromosome 2, from which we analyzed eleven markers in greater detail (**Figure 3**; **Table 1**). The greatest effect size for Peak Fr1 was observed for the marker Rh12GR_62784_393 (β=0.378), and for Peak Fr2, the marker RhMCRND_12686_297 (β= -0.358) [0, 1] presented the greatest effect size. Previous studies have also identified markers for fragrance on chromosomes 2 and 3 (Schulz et al., 2021), and the marker RhMCRND_11924_839 (named Rh_Fr_SNP139K in Schulz et al. (2021)) on chromosome 2 was also found in our analysis. This marker, along with Rh_FR_SNP201K on chromosome 3, serves as a reference in the validation panel.

*Petal: length*

The analysis of petal length revealed a single peak on chromosome 2 (71–72 Mb) (**Figure 3**). On the basis of this peak, five markers were selected for verification. Additionally, in the subanalysis of the new garden rose panel, an additional peak on chromosome 7 (3–4 Mb) was detected (**Supplementary Figure 3**) containing eight significant markers. From this peak, four markers were chosen for validation based on the genomic positions (one marker was selected with the lowest position and one marker with the highest position as well as two from the middle region). The maximum effect size was |β|=2.918 for the marker RhK5_12478_1400 on chromosome 2 (**Table 1**). Interestingly, the marker RhMCRND_6741_1060 was significant for both ‘Petal: length’ and ‘Flower: fragrance’. Additionally, the reference marker on chromosome 5 (Rh_PL_SNP49K) based on Schulz et al. (2021) was also selected for validation.

### 3.3 Validation of associated markers

To validate the marker-trait associations identified in the initial GWAS, selected markers were converted into PACE markers and analyzed in an independent population of 190 rose varieties different from those published previously by Schulz et al. (2021). As phenotypes were not available for all plants for all traits and since not all allele dosage groups could be determined with certainty, the actual number of genotypes with both genetic and phenotypic data ranged from 108 to 182 (**Supplementary Table 3**). The validation experiments revealed that of the 59 markers tested, 26 markers again displayed significant associations with the target traits (p value < 0.05), while seven markers could not be evaluated with statistical certainty because of PACE fluorescence signals. In total, some of the significantly associated markers were validated for six of the eight traits analyzed.

*Young shoot: anthocyanin coloration*

**Figure 4:**
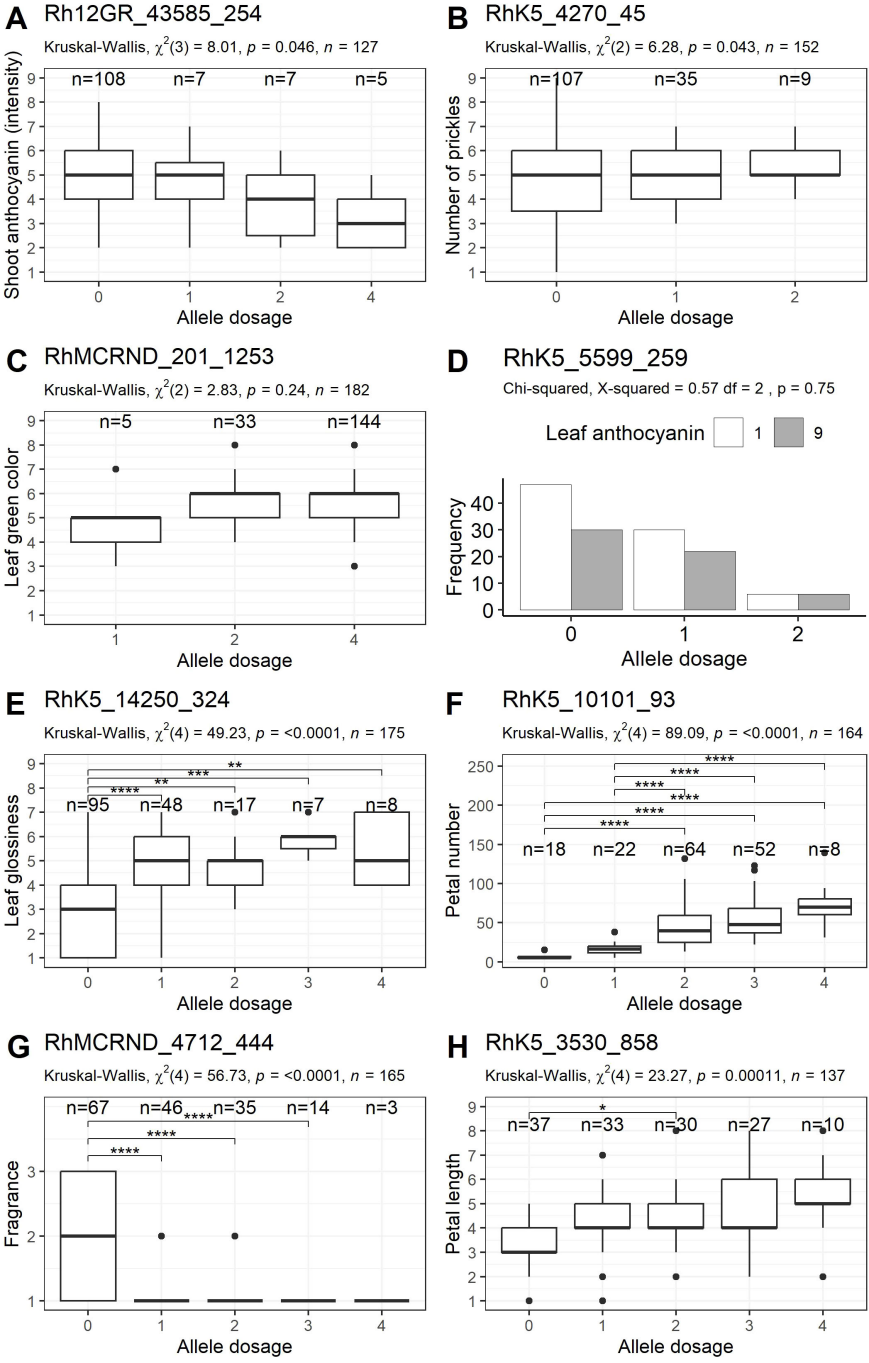
Validation of markers associated with eight phenotypic traits (one example for each trait) in a group of 190 independent cultivars. **(A)** Young shoot: intensity of anthocyanin coloration, **(B)** Stem: number of prickles, **(C)** Leaf: intensity of green color (upper side), **(D)** Leaf: anthocyanin coloration, **(E)** Leaf: glossiness of upper side, **(F)** Flower: number of petals, **(G)** Flower: fragrance, **(H)** Petal: length. The allele dosages are plotted along the x-axis, and the ranges of the phenotypic scores are shown on the y-axis (except for **D**). For **(D)**, the frequency of absent (1) or present (9) leaf anthocyanin is shown on the y-axis. The results of comparisons between individual dosage groups are indicated by brackets, and p values are symbolized by asterisks (*p<0.05, **p<0.01, ***p<0.001, ****p<0.0001).

For this trait, seven significant markers were found in the GWAS analysis, two of which could not be evaluated with statistical certainty in the PACE assay. Among the remaining five markers, only the marker Rh12GR_43585_254 reached overall significance in the validation panel, with a p value of 0.0458 and an effect size of η^2^ = 0.041, although no pairwise differences could be detected (**Supplementary Table 3**; **Figure 4A**). For this marker, 85% of the tested varieties presented allele dosage group 0.

*Stem: number of prickles*

Among the 12 markers developed for this trait, the PACE results for the marker RhK5_3587_1335 could not be analyzed with statistical certainty. Among the 11 markers tested, two markers, RhK5_4270_45 (p value=0.0433) and RhMCRND_2230_1005 (p value=0.00678), were significantly associated with small to moderate effect sizes (**Supplementary Table 3**). As the marker RhMCRND_2230_1005 did not show a genetically meaningful pattern in the associations of the allele dose groups with the trait (scores for allele dose groups 1 and 3 were significantly higher than those for allele dose group 2), only the marker RhK5_4270_45 remained, which only showed overall significance on the basis of a Kruskal–Wallis test (**Figure 4B**).

*Leaf: intensity of green colourcolor on the upper side*

For this trait, four of the seven markers could be analyzed with statistical certainty in the PACE assay. None were significant (**Supplementary Table 3**). The marker RhMCRND_201_1253 showed a trend towards lower values in allele dosage group 1; however, this group contained only five observations (**Figure 4C**).

*Leaf: anthocyanin coloration*

Among the two markers that were developed for the trait ‘Leaf: anthocyanin coloration’, only one marker reached significance in our GWAS analysis. In the validation panel, none of these markers demonstrated significant associations with the trait (**Supplementary Table 3**). However, the marker RhK5_5599_259 displayed a tendency for genotypes with an allele dosage of two to exhibit higher median anthocyanin coloration (**Figure 4D**).

*Leaf: glossiness of upper side*

Two significant markers (Rh12GR_4274_338 and RhK5_14250_324) were evaluated for this trait. Both markers also showed significant associations in the validation panel, with effect sizes of η^2^ = 0.314 and η^2^ = 0.266, respectively, which were greater than those in the association panel (η^2^ = 0.194 and 0.174, respectively) (**Table 1**, **Supplementary Table 3**). For the marker RhK5_14250_324, 55% of the varieties presented allele dosage group 1, which was associated with lower leaf glossiness values (**Figure 4E**).

*Flower: number of petals*

For this trait, five new markers were tested (**Table 1**). Each showed significant associations within the validation panel under the additive model, with large effect sizes ranging from η² = 0.315 to 0.361 for peak PN1 and from η² = 0.228 to 0.535 for peak PN2 (**Supplementary Table 3**). Compared with the validation panel, the effect sizes were smaller in the association panel, ranging from η² = 0.225 to 0.278 for peak PN1 and from η² = 0.156 to 0.348 for peak PN2 (**Table 1**). The marker with the greatest effect size in the validation panel was RhK5_10101_93, with an effect size of η² = 0.535 (**Figure 4F**). In addition to the markers developed in the GWAS, a marker from Hibrand Saint-Oyant et al. (2018), namely, RhK5_4359_382, and a marker from Schulz et al. (2021), namely, Rh_PN_SNP6K, were used as reference markers for comparison with the new markers. Rh_PN_SNP6K did not show significant associations in our validation panel, while RhK5_4359_382 had a high effect size (η² = 0.423); however, this value was lower than that of the best newly developed markers.

*Flower: fragrance*

On the basis of the GWAS results, ten previously unpublished markers for two loci were developed for the trait ‘Flower: fragrance’ (**Table 1**). The marker RhMCRND_13639_80 could not be evaluated with statistical certainty in the PACE assay. All of the remaining nine markers tested exhibited significant associations in the independent validation panel (**Supplementary Table 3**). The markers for peak Fr1 (Rh12GR_62784_393 and Rh12GR_53908_964) were significantly associated with moderate to large effect sizes (η^2^ = 0.093 and 0.231, respectively). For peak Fr2, the effect sizes of the markers were all as large as those in the association panel (**Table 1**), ranging from η^2^ = 0.216 for RhMCRND_12686_297 to η^2^ = 0.33 for RhMCRND_4712_444, indicating a strong dominant effect (**Figure 4G**). In addition, two reference markers from Schulz et al. (2021) were tested. The reference marker from chromosome 2, Rh_FR_SNP139K, showed significant associations with effect sizes of η^2^ = 0.307.

*Petal: length*

For this trait, nine markers were validated from our GWAS analysis, along with one marker from Schulz et al. (2021), Rh_PL_SNP49K from chromosome 5, which was used as a reference marker (**Table 1**). All four markers from chromosome 7 were significant, reaching moderate to large effect sizes from η^2^ = 0.104 to η^2^ = 0.153, which were lower than the effects in the association panel (η^2^ = 0.311 to 0.373) (**Supplementary Table 3**, **Table 1**). The marker with the greatest effect size in the association panel, RhK5_3530_858, also had a large effect in the validation panel (**Figure 4H**). None of the five markers from chromosome 2 were significant in the validation panel. In contrast, the reference marker from chromosome 5, Rh_PL_SNP49K, was significant, with an effect size of η^2^ = 0.047.

### 3.4 Combination of markers from different loci

The additive effect of genetic variation for complex traits influenced by more than one locus can contribute significantly to the explanation of phenotypic variation. We investigated whether the combination of two genetic loci significantly associated with the traits ‘Flower: number of petals’ and ‘Flower: fragrance’ led to an increase in effect sizes that exceeded the individual effect sizes of each individual marker for each of the two traits.

**Figure 5:**
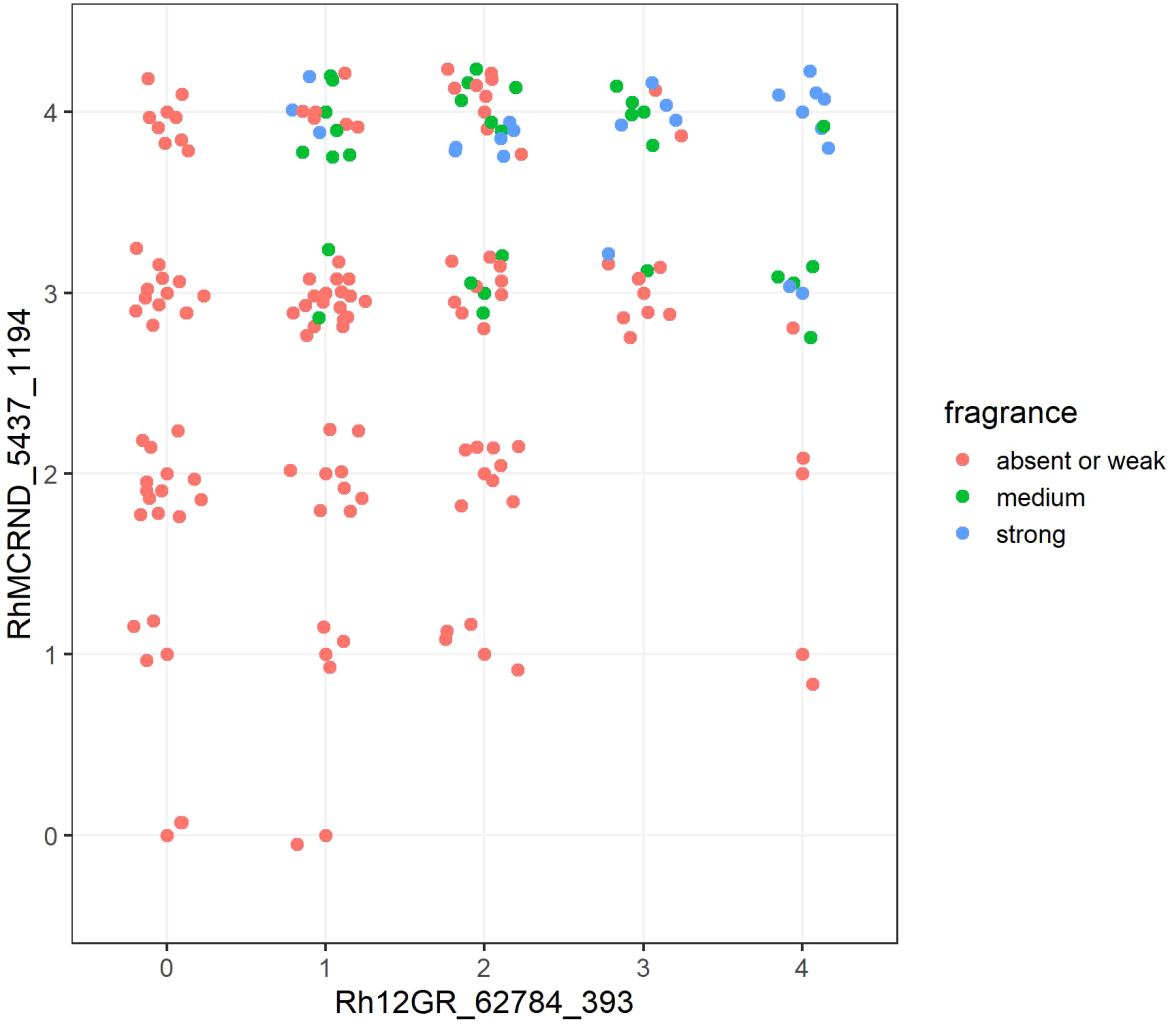
The combination of two markers significantly associated with fragrance in combination with all observed allele dosage groups for each marker. The x axis shows the allele dosage groups for the marker Rh12GR_62784_393 (chromosome 2, peak Fr1), and the y axis shows the allele dosage groups for the marker RhMCRND_5437_1194 (chromosome 2, peak Fr2). The fragrance intensity score is color coded for scores ‘absent or weak’ (1), ‘medium’ (2) or ‘strong’ (3). Each dot shown in the graph represents one genotype of the independent panel.

For the trait ‘Flower: fragrance’, the analysis revealed that the combination of two markers, each representing one of the two associated loci on chromosome 2, resulted in an increase in the effect size for all the markers from Peak Fr2 combined with Rh12GR_62784_393 from peak Fr1 (**Supplementary Table 4**). Interestingly, the combination of the best markers from each of the two loci, RhMCRND_4712_444 (η^2^ = 0.33) and Rh12GR_62784_393 (η^2^ = 0.231), had the second greatest effect size (η^2^ = 0.437). The combination of RhMCRND_5437_1194 (η^2^ = 0.276) and Rh12GR_62784_393 (η^2^ = 0.231) had the greatest effect size with η^2^ = 0.470. In **Figure 5**, allele dosage groups for both markers associated with fragrance are plotted against each other, and the increase in fragrance intensity with increasing allele dosages for each of the favoring alleles is graphically displayed.

When markers for the trait ‘Flower: number of petals’ from two different loci were combined, the effect sizes increased for all the markers except for the combination with RhMCRND_13217_328 from peak PN2 (**Supplementary Table 5**). The greatest effect size (η^2^ = 0.601) was achieved by the combination of the markers RhMCRND_10097_334 (η^2^ = 0.315) and RhK5_10101_93 (η^2^ = 0.535).

## 4 Discussion

### 4.1 GWAS in roses

Following its successful application in human genetics, GWAS has been established as a standard method for the analysis of quantitative traits in both model plants and crops (Alseekh et al., 2021). In recent years, GWAS analyses have been further enhanced by the increasing availability of high-throughput marker systems such as SNP chips and NGS-based methods such as RAD-Seq and GBS (Pavan et al., 2020). GWAS have therefore also been applied to a number of ornamental species, such as chrysanthemums, orchids and roses (Smulders et al., 2019; Sumitomo et al., 2019; Hsu et al., 2022). In roses, most analyses have been carried out in very small association panels of fewer than 100 plants. For the development of markers with the potential to be applied in commercial breeding, this may be sufficient, as only QTLs with major effects will be of interest in marker-assisted breeding. However, when genomic intervals around causative genes are delineated, small panels do not provide a sufficient level of resolution in plant research.

We have extended our previous GWAS studies in roses by analyzing new traits (prickles, leaf color, leaf size, leaf/shoot anthocyanin coloration and leaf glossiness). Furthermore, we have improved the resolution of our analyses for traits already analyzed in previous studies (Hibrand Saint-Oyant et al., 2018; Schulz et al., 2021) by using a larger panel size of 285 genotypes and software developed for tetraploids. The traits analyzed here are part of the UPOV guidelines for Distinctness, Uniformity and Stability (DUS) testing according to Test Guideline TG/11/8 (https://www.upov.int/edocs/tgdocs/en/tg011.pdf) and might therefore be of interest in analyses of the genetic basis underlying these traits. These data add to the knowledge on the inheritance of important ornamental characteristics in roses. This information can be used to estimate the complexity of the traits under study.

For all six traits for which we found marker-trait associations and verified them in independent panels, we detected one to three major QTLs. However, the small size of our association panel does not allow the detection of small-effect QTLs contributing to phenotypic variation. This is supported by the effect sizes, which explain 8% to 19% (using the best marker per locus) of the phenotypic variation in the population we studied (**Supplementary Table 6**).

The trait for which we found no marker-trait associations (Leaf: size) may be influenced by a larger number of genes, each with a small effect size that escaped our detection, and/or express lower heritability with a greater influence of the environment on the trait. However, the latter explanation is unlikely, as high heritability is a prerequisite for a trait to be considered for UPOV classification.

For the two traits where marker-trait associations were detected but could not be validated (‘Leaf: anthocyanin coloration’ and ‘Leaf: intensity of green color on the upper side’), either a greater environmental influence may have acted on the phenotypes or the selection of the genotypes in the association panels was not sufficiently representative of garden roses in general. One reason could be that one-third of the panel consisted of cut roses. This highlights the need for validation procedures for GWAS studies with low to moderate panel sizes, as still used in other ornamental crops (Sumitomo et al., 2019; Hsu et al., 2022).

### 4.2 Validation of the GWAS results

As association studies often suffer from confounding effects such as population structure (see discussion above), stringent controls are necessary to exclude false-positive associations. Therefore, the first step was to exclude any associated markers that were not part of a marker peak that included multiple markers from most analyses. This separates associated markers from the background noise of the majority of the markers. The rationale behind this idea is that with more than 30,000 markers analyzed in the GWAS, it is not expected that individual markers will be associated with traits but rather that groups of markers in genomic regions will be associated with traits owing to linkage disequilibrium.

Much larger association panels with strictly controlled population structure would be one way to reduce putative artefacts but are extremely resource intensive for both phenotyping and genotyping. Using a less resource-intensive strategy, we validated our results by converting the SNP chip markers to PACE markers (Maydell, 2023), a PCR-based method, and analyzing them in an independent population. The reproducibility of the associations in the independent population suggests that the identified markers are not only specific to the original population studied but may also be suitable for broader application in rose breeding. Notably, for the three traits analyzed previously (petal number, petal size and fragrance), the validation panel used in this study is completely independent of the validation panel used in Schulz et al. (2021), thus further increasing the population in which the associated loci have been validated. In another previously published study, we have shown that markers that pass such a validation process are useful for selecting the optimal parental genotypes for certain traits in commercial breeding programs (Schulz et al., 2023), further supporting their true association with phenotypes. However, with this strategy (using smaller sample sizes and validate the results), it is unlikely to find rare alleles due to low statistical power. An even larger sample size is particularly important for autopolyploids like roses in order to find rare alleles. This is because more allele dose groups reduce the effective number of markers that can be analyzed with sufficient allele dose resolution in genome-wide analyses.

### 4.3 Genetic complexity of the traits studied

The marker-trait associations we detected for the eight traits indicate a prominent contribution of a few major QTLs to each of the traits, as indicated by only one to five significantly associated loci detected for each trait. It is likely that several other QTLs with minor effects remained undetected in our study because of the limited panel size or suboptimal phenotyping due to the use of the scale defined to the UPOV test guideline in the frame of granting Plant breeders rights. In addition, we cannot exclude the possibility that the composition of the panel may obscure some marker-trait associations that may only be present in certain subpopulations of roses not represented in our panel of 285 genotypes. Finally, although our panel consisted of randomized replicate trials, the environmental effects caused by the collection of data over different seasons cannot be completely excluded. Furthermore, roses are autotetraploid, which means that they have more complex inheritance patterns associated with increased genetic complexity. For genotyping with SNP chips or other fluorescence based methods which are based on biallelic SNP detection that genetic information at polymorphic sites is either lost by focusing on only two alleles or that detection of all possible alleles is very resource intensive.

#### 4.3.1 Anthocyanin coloration of the young shoot

For the anthocyanin coloration of young shoots, we identified single markers in different regions on chromosomes 2 and 4, with our validated marker located on chromosome 2 at 61.81 Mb. Research has suggested that the RcMYB1 gene plays an important role in controlling anthocyanin biosynthesis for floral color in roses (He et al., 2023). In addition, Rosa rugosa RrMYB12 and RrMYB111 are known to be expressed at low levels in roots and flowers and have been shown to function as transcriptional activators in transactivation activity assays (Shi et al., 2024). Our region is related to MYB101 (chromosome 2, 62.25 Mb in Rosa chinensis), an R2R3 MYB gene that acts as a repressor of anthocyanin biosynthesis in pepper (Liu et al., 2021). These findings highlight the complex regulation of anthocyanin biosynthesis in roses, which may also be influenced by environmental factors such as sucrose levels and ammoniac nitrogen availability (Ram et al., 2011).

#### 4.3.2 Prickle number

Our analysis of prickle number revealed significant loci on chromosomes 3 (45 Mb) and 5 (13–14 Mb). Consistent with our findings, Hibrand Saint-Oyant et al. (2018) identified a QTL for prickle density in the same region on chromosome 3 through QTL analysis in two F1 progenies. Similarly, Crespel et al. (2002) identified major and minor QTLs in linkage group 3 in the vicinity of a single seasonally blooming gene. Notably, a WRKY transcription factor, homologous to Arabidopsis TTG2, is located near this region and is known to regulate prickle density, as its gene transcripts are differentially expressed between prickle and prickless roses (Hibrand Saint-Oyant et al., 2018). However, the markers from this region were not confirmed by our validation panel, suggesting potential differences in genetic backgrounds or environmental influences. Furthermore, previous studies have identified QTLs related to prickle number in linkage groups 2, 4, and 6 (Hibrand-Saint Oyant et al., 2007; Bourke et al., 2018), indicating the complex genetic architecture underlying prickle traits.

#### 4.3.3 Leaf traits: green color, anthocyanin content and glossiness

Leaf color, anthocyanin concentration and glossiness of the upper side are all dominated by a single large-effect QTL, and in the case of glossiness and green color, some additional peaks do not reach the significance threshold. The effect sizes of the major associated markers for these traits each account for approximately 10% of the phenotypic variation, so a complex genetic architecture with additional QTLs with smaller effect sizes is very likely. However, Cheng and Yu (2023) also detected a QTL in the tetraploid population YS on chromosome 4 for anthocyanin coloration in roses, explaining approximately 16% of the phenotypic variation, as well as a second QTL with a similar effect on chromosome 6.

The strongest marker–trait associations are shown for petal number, petal size and floral fragrance, three traits that have already been genetically characterized in several previous studies (Hibrand Saint-Oyant et al., 2018; Schulz et al., 2021).

#### 4.3.4 Petal number

For petal number, both a dominant single locus and an additional QTL have been mapped in several studies in both biparental populations and in association studies in diploids and tetraploids (Debener and Mattiesch, 1999; Dugo et al., 2005; Hibrand-Saint Oyant et al., 2007; Spiller et al., 2011; Bourke et al., 2018; Schulz et al., 2021). The dominant single locus corresponds to the peak at 33 Mb, with an effect size of 19% explained variation. The strong effect of this locus could be influenced by a dominant gene for double flowers that has been mapped exactly to one of the QTL positions on chromosome 3 (Debener and Mattiesch, 1999; Spiller et al., 2011; Hibrand Saint-Oyant et al., 2018). A likely candidate for this gene is the AP2 paralogue in which the binding site of a miRNA is mutated in the double-flower allele of the gene (Hibrand Saint-Oyant et al., 2018). However, conclusive evidence for the function of this gene as the single dominant double-flower locus previously described is still lacking. In close proximity, another QTL was found that cannot be easily separated from the AP2 locus because of its genetic effect of approximately 18%.

#### 4.3.5 Petal length

Our analysis of petal length revealed a single significantly associated locus on chromosome 2 explaining approximately 10% of the phenotypic variation in the association panel. For this trait, additional peaks were identified in a previous study by Schulz et al. (2021), where peaks on chromosomes 1, 2 and 5 had significant effects. However, this study used allele dosage data from only 95 genotypes in a garden rose panel that had been collapsed to fit a diploid model, and the analysis was performed using a different software (TASSEL) designed for diploids. The different results between the detected QTLs in the different subpopulations, with a single peak on chromosome 7 in the new garden rose panel and two significant peaks in the other garden rose panel on chromosomes 2 and 4 but no significant peak in the cut rose panel, show that GWAS analyses are sensitive to the selection of the genotypes for the association panel. However, the fact that we were able to validate markers from the peak on chromosome 7 indicates the robustness of this effect, at least in garden roses.

#### 4.3.6 Fragrance

For fragrance, we focused our analyses on two highly significant loci on chromosome 2, although some minor peaks also occurred on chromosomes 4 and 6. Numerous studies have analyzed the floral scent or fragrance of roses, which is an extremely complex trait consisting of a large number of volatiles that are emitted mainly from petals (Knudsen et al., 2006; Cherri-Martin et al., 2007; Magnard et al., 2015). While research focusing on the biochemistry of scent volatiles has identified a number of genes responsible for volatile synthesis and/or localized the corresponding genes in the genome (Spiller et al., 2010, 2011; Magnard et al., 2015; Shang et al., 2024), some studies have used scores based on scent recognition by humans to phenotype populations that differ in fragrance intensity (Schulz et al., 2021). While Schulz et al. (2021) detected a locus with a large effect at the end of chromosome 2 in garden roses, our analysis including cut roses detected a second peak on chromosome 2 at approximately 41 Mb. This is obviously an effect of the composition of the association panel and needs to be considered for all traits for which subpopulations differ, such as garden and cut roses. In the case of the second peak, we speculate that this genomic region plays an important role in the production of the very light scent observed in some genotypes of cut roses, which generally emit less scent than garden roses. Studies localizing individual components of fragrance in the rose genome have mapped geranylacetate and a member of the BEAT enzyme family, which is responsible for the formation of volatile esters from alcohols (Spiller et al., 2010). As volatile esters are major compounds of fragrance in roses, this may explain at least part of the effect of this locus at the end of chromosome 2 on fragrance. The RhNUDX1 gene, which encodes a Nudix hydrolase involved in the biosynthesis of monoterpene alcohols and is therefore important for the scent of roses (Magnard et al., 2015), is located around 59.6 Mb on chromosome 2, which is outside the two major QTLs identified in our study.

### 4.4 Can we speculate about candidate genes?

We mostly refrain from speculating on the nature of the causal genes underlying the QTLs we found, as the resolution of our marker–trait associations does not allow the identification of only a few candidates but rather encompasses larger genomic intervals with numerous gene models. Reducing this to smaller candidate sets would first require functional analyses, which was beyond the aim of our study, which focused on detecting and validating markers associated with the important traits for their use in MAS and determining the genetic complexity of trait inheritance. However, the fact that we detected highly reproducible markers, e.g., fragrance and petal number, in several rose populations indicates that these loci play important general roles in specific traits in roses.

### 4.5 How can we apply the data in rose breeding and genetics?

A general problem in the application of markers for single traits and QTLs is the range of genotypes in which they might detect the same effects as the populations from which they were derived. It is believed that markers derived from GWAS represent a wider range of genotypes than those derived from studies involving biparental populations and should therefore have wider applicability. If properly validated, these markers could be successfully used to analyze many different genotypes from commercial breeding programs. In a previous study by Schulz et al. (2023), it is shown that markers for petal number, petal size and fragrance derived from the garden rose association panel accurately predicted the distribution of phenotypes in a cut rose breeding program using marker-assisted selection (MAS) of parents. The offspring of parental combinations with contrasting allele dosages, resulting in either higher or lower expression of these phenotypic traits, presented significant differences in trait distribution. Here, we demonstrated that in addition to the markers/loci found by Schulz et al. (2021) and Hibrand Saint-Oyant et al. (2018), improved markers with increased effect sizes can be selected close to the original markers. In addition, we developed markers for additional traits, such as the glossiness of the upper leaf side, which may be useful in the selection of special trait combinations in commercial breeding.

An investigation of the combined effects of two markers revealed an increased effect size, indicating additive effects and possible interactions between the genetic loci. These findings may be useful in the development of optimized breeding strategies and may help to increase breeding progress through targeted marker combinations.

## Data Availability

The datasets presented in this study can be found in online repositories. The names of the repository/repositories and accession number(s) can be found below: https://doi.org/10.6084/m9.figshare.28391357, https://doi.org/10.6084/m9.figshare.28388243, https://doi.org/10.6084/m9.figshare.28358720.
