# Supplementary material for: Beyond bloom: validated marker–trait discovery for polyploid roses via GWAS
Source: Front Plant Sci. 2025 May 13;16:1591861. doi: 10.3389/fpls.2025.1591861 (PMC12106473; doi:10.3389/fpls.2025.1591861)
Supplement: Supplementary file 1 [file DataSheet1.docx]

Supplementary Material

“Beyond Bloom: Validated marker-trait discovery for polyploid roses via GWAS”


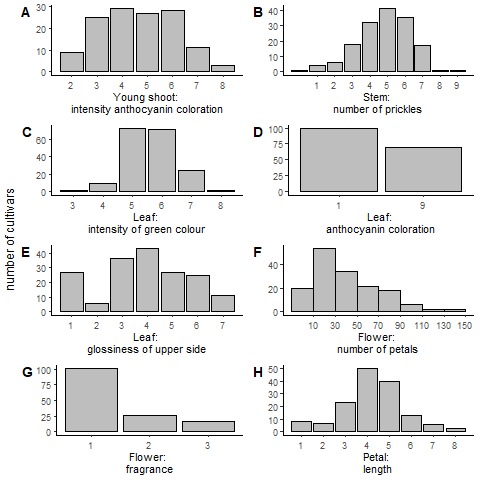


**Supplementary Figure 1.** Distribution of the mean for Young shoot: intensity of anthocyanin coloration (A), Stem: number of prickles (B), Leaf: intensity of green colour (C), Leaf: anthocyanin coloration (D), Leaf: glossiness of upper side (E), Flower: number of petals (F), Flower: fragrance (G) and Petal: length (H) in the independent panel.


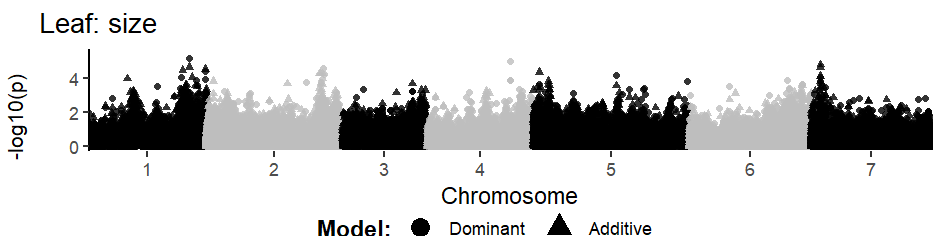


**Supplementary Figure 2.** Manhattan plots for the non-significant marker-trait-associations of rose ornamental traits in a set of 285 garden and cut roses. The x-axis indicates the genomic position of the markers and the y-axis indicates the negative logarithmic p-value (-log10 (p)) for each marker-trait association. Each dot represents a single SNP.


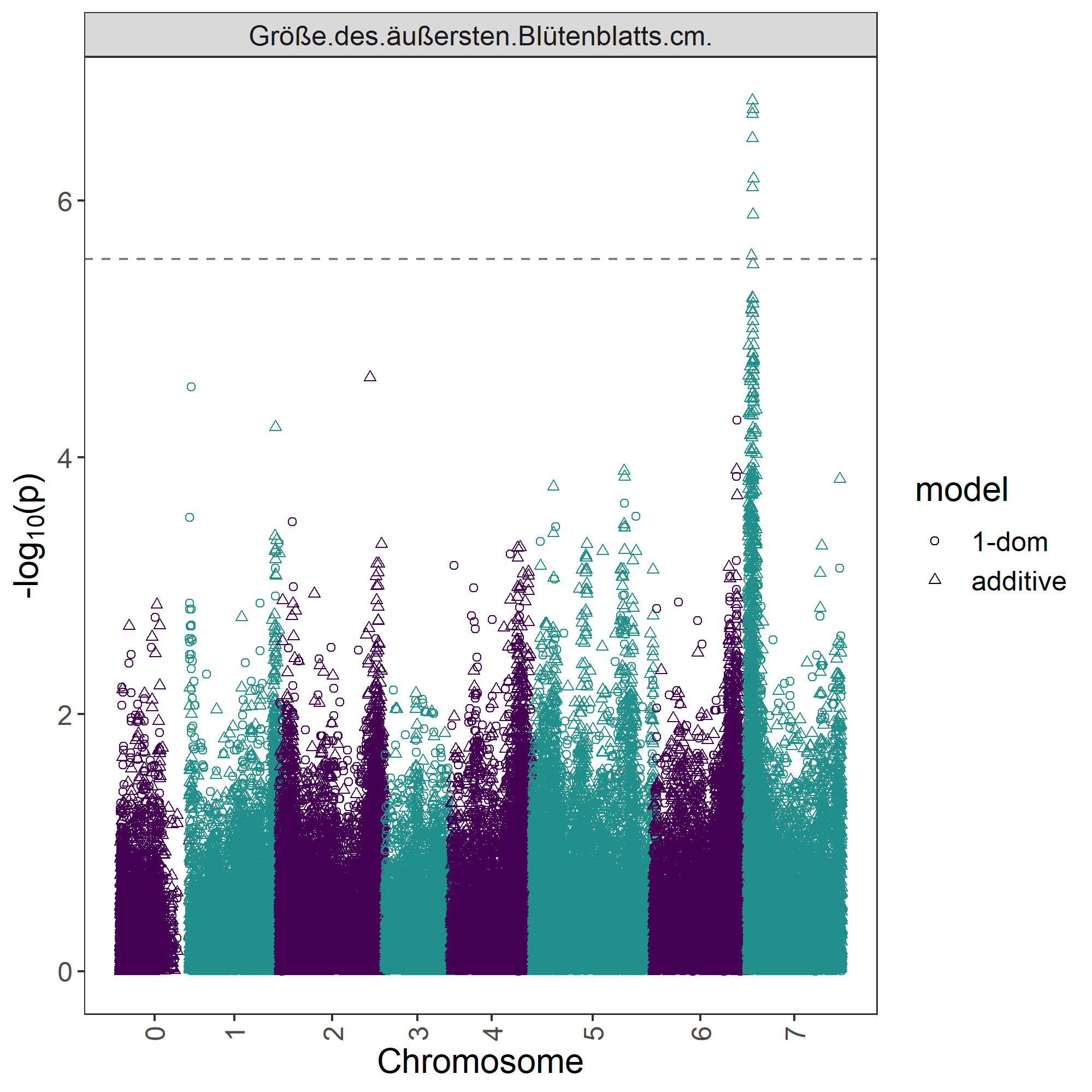


Supplementary Figure 3. Manhattan plot for ‘Petal: length’ in the new garden rose association panel.

**Supplementary Table 1.** Varieties and cultivars in the ‘new garden rose’ association panel.

| **Code** | **Variety/cultivar** | **Breeder** |
| --- | --- | --- |
| AS001 | Korbehati | W. Kordes' Söhne Rosenschulen GmbH & Co. KG |
| AS002 | Korrahibe | W. Kordes' Söhne Rosenschulen GmbH & Co. KG |
| AS003 | Korchakon | W. Kordes' Söhne Rosenschulen GmbH & Co. KG |
| AS004 | Korcoluma | W. Kordes' Söhne Rosenschulen GmbH & Co. KG |
| AS005 | Korstarnow | W. Kordes' Söhne Rosenschulen GmbH & Co. KG |
| AS006 | Korplunblo | W. Kordes' Söhne Rosenschulen GmbH & Co. KG |
| AS007 | Korgeleflo | W. Kordes' Söhne Rosenschulen GmbH & Co. KG |
| AS008 | Korwedesi | W. Kordes' Söhne Rosenschulen GmbH & Co. KG |
| AS009 | Korzwanlio | W. Kordes' Söhne Rosenschulen GmbH & Co. KG |
| AS010 | TAN07890 | Rosen Tantau Vertrieb GmbH & Co. KG |
| AS011 | TAN15603 | Rosen Tantau Vertrieb GmbH & Co. KG |
| AS012 | Haramity | Harkness |
| AS013 | Auscot | David Austin Roses Ltd. |
| AS014 | Bokrapolo | De Ruiter Innovations B.V. |
| AS015 | Tanabamar | Rosen Tantau Vertrieb GmbH & Co. KG |
| AS016 | Old Blush | unknown |
| AS017 | 91/100-5 | own crossings, Debener |
| AS018 | Macrexy | McGredy Roses International |
| AS019 | 07/57-5a | own crossings, Debener |
| AS020 | Clasysnow | W. Clayworth |
| AS021 | 07/57-61a | own crossings, Debener |
| AS022 | 07/57-64a | own crossings, Debener |
| AS023 | Korcolipas | W. Kordes' Söhne Rosenschulen GmbH & Co. KG |
| AS024 | TAN15555 | Rosen Tantau Vertrieb GmbH & Co. KG |
| AS025 | Meilangele | MEILLAND International |
| AS026 | Kortrameilo | W. Kordes' Söhne Rosenschulen GmbH & Co. KG |
| AS027 | TAN98130 | Rosen Tantau Vertrieb GmbH & Co. KG |
| AS028 | Tanotax | Rosen Tantau Vertrieb GmbH & Co. KG |
| AS029 | TAN05244 | Rosen Tantau Vertrieb GmbH & Co. KG |
| AS030 | TAN05415 | Rosen Tantau Vertrieb GmbH & Co. KG |
| AS031 | TAN08387 | Rosen Tantau Vertrieb GmbH & Co. KG |
| AS032 | NOA70896 | Noack Rosen |
| AS033 | NOA150097 | Noack Rosen |
| AS034 | NOA130400 | Noack Rosen |
| AS035 | NOA83100B | Noack Rosen |
| AS036 | NOA195097 | Noack Rosen |
| AS037 | NOA75800 | Noack Rosen |
| AS038 | NOA97400A | Noack Rosen |
| AS039 | NOA23104 | Noack Rosen |
| AS040 | NOA16071 | Noack Rosen |
| AS041 | NOA51071 | Noack Rosen |
| AS042 | NOA13702A | Noack Rosen |
| AS043 | NOA20054 | Noack Rosen |
| AS044 | Korgretaum | W. Kordes' Söhne Rosenschulen GmbH & Co. KG |
| AS045 | Korditwol | W. Kordes' Söhne Rosenschulen GmbH & Co. KG |
| AS046 | Taninaso | Rosen Tantau Vertrieb GmbH & Co. KG |
| AS047 | Korsilu06 | W. Kordes' Söhne Rosenschulen GmbH & Co. KG |
| AS048 | Korfruisala | W. Kordes' Söhne Rosenschulen GmbH & Co. KG |
| AS049 | Korfliaumi | W. Kordes' Söhne Rosenschulen GmbH & Co. KG |
| AS050 | Korfizzlem | W. Kordes' Söhne Rosenschulen GmbH & Co. KG |
| AS051 | Kaiser von Lautern | Karl Heinz Michler |
| AS052 | Korhubkah | W. Kordes' Söhne Rosenschulen GmbH & Co. KG |
| AS053 | Koroutofko | W. Kordes' Söhne Rosenschulen GmbH & Co. KG |
| AS054 | PEACLOE | Colin A. Pearce |
| AS055 | Korhumalex | W. Kordes' Söhne Rosenschulen GmbH & Co. KG |
| AS056 | Kormolibla | W. Kordes' Söhne Rosenschulen GmbH & Co. KG |
| AS057 | Kormonali | W. Kordes' Söhne Rosenschulen GmbH & Co. KG |
| AS058 | Koradigel | W. Kordes' Söhne Rosenschulen GmbH & Co. KG |
| AS059 | Korhulth002 | W. Kordes' Söhne Rosenschulen GmbH & Co. KG |
| AS060 | Kordiagraf | W. Kordes' Söhne Rosenschulen GmbH & Co. KG |
| AS061 | Kormelpea | W. Kordes' Söhne Rosenschulen GmbH & Co. KG |
| AS062 | Korcharblu | W. Kordes' Söhne Rosenschulen GmbH & Co. KG |
| AS063 | Lenalbi | Lens Roses |
| AS064 | Meihadrall | MEILLAND International |
| AS065 | Meipelmel | MEILLAND International |
| AS066 | Adamariat | NIRP International S.A. |
| AS067 | Delagak | Delbard |
| AS068 | Chewrocko | Warners Roses Ltd |
| AS069 | Sanders White Rambler | Sander & Sons |
| AS070 | Radbrite | Star Roses and Plants |
| AS071 | Tanledolg | Rosen Tantau Vertrieb GmbH & Co. KG |
| AS072 | Korelifur | W. Kordes' Söhne Rosenschulen GmbH & Co. KG |
| AS073 | Korsilu07 | W. Kordes' Söhne Rosenschulen GmbH & Co. KG |
| AS074 | Korelpusch | W. Kordes' Söhne Rosenschulen GmbH & Co. KG |
| AS075 | TAN99176 | Rosen Tantau Vertrieb GmbH & Co. KG |
| AS076 | MEHV9601 | Bernard Mehring |
| AS077 | TAN12628 | Rosen Tantau Vertrieb GmbH & Co. KG |
| AS078 | Tanrezlaw | Rosen Tantau Vertrieb GmbH & Co. KG |
| AS079 | Korlitare | W. Kordes' Söhne Rosenschulen GmbH & Co. KG |
| AS080 | Korgellan | W. Kordes' Söhne Rosenschulen GmbH & Co. KG |
| AS081 | TAN97274 | Rosen Tantau Vertrieb GmbH & Co. KG |
| AS082 | TAN05281 | Rosen Tantau Vertrieb GmbH & Co. KG |
| AS083 | Tanallepa | Rosen Tantau Vertrieb GmbH & Co. KG |
| AS084 | TAN00847 | Rosen Tantau Vertrieb GmbH & Co. KG |
| AS085 | Mermaid | Paul |
| AS086 | Honey Bee | unknown |
| AS087 | TAN04341 | Rosen Tantau Vertrieb GmbH & Co. KG |
| AS088 | TAN04608 | Rosen Tantau Vertrieb GmbH & Co. KG |
| AS089 | TAN09414 | Rosen Tantau Vertrieb GmbH & Co. KG |
| AS090 | TAN09505 | Rosen Tantau Vertrieb GmbH & Co. KG |
| AS091 | Taniripsa | Rosen Tantau Vertrieb GmbH & Co. KG |
| AS092 | TAN03525 | Rosen Tantau Vertrieb GmbH & Co. KG |
| AS093 | *R.foetida* 'Soleil d´Or' | Pernet-Ducher |
| AS095 | *Rosa majalis* 93/09-01 | own crossings, Debener |
| AS096 | *R. spinosissima* |  |

Supplementary Table 2. PACE Primer Sequences. Markers in bold were previously described in publications by Hibrand Saint-Oyant et al. (2018) and Schulz et al. (2021). The markers in red are significant for two separate phenotypic traits.

| **Trait** | **Marker** | **Allele-specific primer 1** | **Allele-specific primer 2** | **Common, Reverse primer** |
| --- | --- | --- | --- | --- |
| Young shoot: intensity of anthocyanin coloration | RhK5_17994_176 | GAAGGTGACCAAGTTCATGCTGAGTCTGTACGTGTCGGTGACA | GAAGGTCGGAGTCAACGGATTAGTCTGTACGTGTCGGTGACG | CATTGCTCCTTTTCCTCCCAACCAA |
|  | Rh12GR_43585_254 | GAAGGTGACCAAGTTCATGCTTCTGAAAAGGAAATATGGGGAATGAT | GAAGGTCGGAGTCAACGGATTCTGAAAAGGAAATATGGGGAATGAC | CTTTTCTGCAAACAACAGAGGGTCTTATA |
|  | Rh12GR_30029_334 | GAAGGTGACCAAGTTCATGCTACCCCGACGATAGGATCAGACT | GAAGGTCGGAGTCAACGGATTCCCGACGATAGGATCAGACG | TCCACCTGAGCATCCCTGAAAACAA |
|  | RhK5_1892_483 | GAAGGTGACCAAGTTCATGCTGCAAATAAGAATGTTCACCTCCT | GAAGGTCGGAGTCAACGGATTCTGCAAATAAGAATGTTCACCTCCG | GGAGTTCCTATCAATGGGTTATCAGTATT |
|  | Rh12GR_38290_1803 | GAAGGTGACCAAGTTCATGCTAGCTACCAAGTTGAGATATACTCATCT | GAAGGTCGGAGTCAACGGATTGCTACCAAGTTGAGATATACTCATCC | AGGAGACCCGGAAAGCCTCCAA |
|  | Rh12GR_19603_664 | GAAGGTGACCAAGTTCATGCTCACTCTTGTCTTTGAAATATGACCTTTCT | GAAGGTCGGAGTCAACGGATTCTCTTGTCTTTGAAATATGACCTTTCC | CGGAGAAGCCAATATACATAGTTTCGTTA |
|  | RhMCRND_5216_974 | GAAGGTGACCAAGTTCATGCTCAAAGCGATGAAACAAGTTTCACAGA | GAAGGTCGGAGTCAACGGATTCAAAGCGATGAAACAAGTTTCACAGC | CGGCAGCTTTAACAGCAAAACTCAATATT |
| Stem: number of prickles | RhMCRND_15645_551 | GAAGGTGACCAAGTTCATGCTTCCAACTATAGCCTAGTCTGCCTT | GAAGGTCGGAGTCAACGGATTCCAACTATAGCCTAGTCTGCCTC | CTTTGCACTACAAGTAACTGAAATTGTGTT |
|  | RhK5_1809_431 | GAAGGTGACCAAGTTCATGCTGTCTGTCATCTTCGTGTTGATTGATTAA | GAAGGTCGGAGTCAACGGATTCTGTCATCTTCGTGTTGATTGATTAG | GCTGCGAATTGCATACAAACTCGGTT |
|  | RhK5_432_2321 | GAAGGTGACCAAGTTCATGCTTCAGGCTGTCGTATTTCTACTTCT | GAAGGTCGGAGTCAACGGATTCAGGCTGTCGTATTTCTACTTCC | GACTACAACTGCCATTGTTCTGAAGAATA |
|  | RhMCRND_4282_2838 | GAAGGTGACCAAGTTCATGCTCCTGTGAAACCAGGCGTTCTT | GAAGGTCGGAGTCAACGGATTCCTGTGAAACCAGGCGTTCTC | CACTGGCAAAGGATGTGGACTTTGAA |
|  | RhK5_5501_1135 | GAAGGTGACCAAGTTCATGCTATTAAGAATAGGAAAAACGAATAGCGTGT | GAAGGTCGGAGTCAACGGATTAAGAATAGGAAAAACGAATAGCGTGG | CGACGGCGATTCGCGTTCTCTT |
|  | RhMCRND_12154_179 | GAAGGTGACCAAGTTCATGCTGGTTTCGGCGGCTTCCCCAA | GAAGGTCGGAGTCAACGGATTGTTTCGGCGGCTTCCCCAG | GACGATCAGCGATTGGTTTCCGATA |
|  | RhK5_4270_45 | GAAGGTGACCAAGTTCATGCTCTCTGGTTGCACTCTCTTAACCA | GAAGGTCGGAGTCAACGGATTCTCTGGTTGCACTCTCTTAACCG | TTGTTGTCAGCACCTAAATCCGGATAATT |
|  | RhMCRND_2230_1005 | GAAGGTGACCAAGTTCATGCTTTCGAACATGATTACCATTAAGCATAGT | GAAGGTCGGAGTCAACGGATTCGAACATGATTACCATTAAGCATAGG | CTACTCCTCCCGCTTGATTTCAGAA |
|  | RhK5_3587_1335 | GAAGGTGACCAAGTTCATGCTGTCCTTTCTCAATTCAACACAACTCTTA | GAAGGTCGGAGTCAACGGATTCCTTTCTCAATTCAACACAACTCTTG | ACCATCCATTCCTGGTATTGTAGTTGAA |
|  | RhMCRND_33463_124 | GAAGGTGACCAAGTTCATGCTAAACTCTGCACCAACTGCTCACAT | GAAGGTCGGAGTCAACGGATTACTCTGCACCAACTGCTCACAC | GCTATGGAGTTAGGGAGCTTTGTTTTATT |
|  | RhK5_12076_566 | GAAGGTGACCAAGTTCATGCTTGGCAGCTGCTGGTCTTGTT | GAAGGTCGGAGTCAACGGATTGGCAGCTGCTGGTCTTGTG | ACATGGTCAAGCAAACGCTGAGGAT |
|  | Rh12GR_16070_248 | GAAGGTGACCAAGTTCATGCTGGAAAAGTTGAACTGATTAAGACATGT | GAAGGTCGGAGTCAACGGATTGGAAAAGTTGAACTGATTAAGACATGC | CAGAGCTTAAAGAGATACAATGCTCCTT |
| Leaf: intensity of green color | BlGF_RhMCRND_201_1253 | GAAGGTGACCAAGTTCATGCTACTTTTAGAAACAGCTGAGCCTGCA | GAAGGTCGGAGTCAACGGATTTTAGAAACAGCTGAGCCTGCC | GGGTAGAAAGTCCTGTAGCATCTGAA |
|  | BlGF_RhMCRND_32494_229 | GAAGGTGACCAAGTTCATGCTAGAGATTGGAGGACTTATCCGATCA | GAAGGTCGGAGTCAACGGATTAGATTGGAGGACTTATCCGATCC | GAGCACCTCCTCCTAATTTGCTCTA |
|  | BlGF_Rh88_48595_614 | GAAGGTGACCAAGTTCATGCTACCTCGTTTTCCCATTCAATTGTACAT | GAAGGTCGGAGTCAACGGATTCCTCGTTTTCCCATTCAATTGTACAC | GTCTACTTGAATCCCTTGTTTTGGAAGTT |
|  | RhK5_8654_792 | GAAGGTGACCAAGTTCATGCTTCCGTTGCCATTTTGTTGTGCACT | GAAGGTCGGAGTCAACGGATTCCGTTGCCATTTTGTTGTGCACC | GTAGGGACTAAAAGGAGGTGCCAAA |
|  | RhK5_16131_601 | GAAGGTGACCAAGTTCATGCTGGATGATTCCAGAAGTGTAGTTCTGA | GAAGGTCGGAGTCAACGGATTGATGATTCCAGAAGTGTAGTTCTGG | CTCTTGTTTACAGTCAGCTTTACCCTTAT |
|  | RhK5_2954_1267 | GAAGGTGACCAAGTTCATGCTAATTTTTTGGCCAAATTGATATGGTTTCTTATA | GAAGGTCGGAGTCAACGGATTTTTGGCCAAATTGATATGGTTTCTTATG | ATGGTTTGGAGAACCTCCAGTCCTA |
|  | RhMCRND_18571_180 | GAAGGTGACCAAGTTCATGCTGGAACTGGTGGTATGTTGATTCT | GAAGGTCGGAGTCAACGGATTGGAACTGGTGGTATGTTGATTCC | GGATATGTCTGCTTCAACAAGAAGAACAA |
| Leaf: anthocyanin coloration | RhK5_5599_259 | GAAGGTGACCAAGTTCATGCTGAGAAGAAGCTGGACGACGACA | GAAGGTCGGAGTCAACGGATTAGAAGAAGCTGGACGACGACG | CATTTCCGTCTTCTTCTTCCGCCAA |
|  | RhMCRND_1644_1712 | GAAGGTGACCAAGTTCATGCTTATATTCAAGCTTTGTTCCTCCTTTGTTA | GAAGGTCGGAGTCAACGGATTATTCAAGCTTTGTTCCTCCTTTGTTG | CCCTTGAGCTTCCCAAAACAGCATA |
| Leaf: glossiness of upper side | Rh12GR_4274_338 | GAAGGTGACCAAGTTCATGCTCAGCCTTAGAGGATTCTGATTCGATA | GAAGGTCGGAGTCAACGGATTAGCCTTAGAGGATTCTGATTCGATG | GCCGAGCTGATTTAAAGCTTCCTGTT |
|  | RhK5_14250_324 | GAAGGTGACCAAGTTCATGCTGGATAGCTCCGGGAATTTTAAAGCT | GAAGGTCGGAGTCAACGGATTGATAGCTCCGGGAATTTTAAAGCC | GTTGAAATTGTCTGATTCCCCTGATACAA |
| Flower: number of petals | Rh12GR_54461_324 | GAAGGTGACCAAGTTCATGCTAATTTTAACTGATTTCCATAGTAATTGAAACCA | GAAGGTCGGAGTCAACGGATTTAACTGATTTCCATAGTAATTGAAACCG | TAAGATTGAATCAAACTAAGGAAGCTCCAA |
|  | RhMCRND_10097_334 | GAAGGTGACCAAGTTCATGCTGAACCTGCATCCAAAAAGGTCTCAA | GAAGGTCGGAGTCAACGGATTAACCTGCATCCAAAAAGGTCTCAC | TTAAGCAGTTAGTGCAGGCTTGTTATGTA |
|  | RhMCRND_13217_328 | GAAGGTGACCAAGTTCATGCTCCGATCGCGAATATTAGCAGGATT | GAAGGTCGGAGTCAACGGATTCGATCGCGAATATTAGCAGGATC | TCGCCGGAAGCGCCTTCTTCAT |
|  | RhMCRND_760_1054 | GAAGGTGACCAAGTTCATGCTGATGTTAATAGGGATGACCTGCCT | GAAGGTCGGAGTCAACGGATTATGTTAATAGGGATGACCTGCCG | CCAACTACAGCCGTCTGCATAACTT |
|  | RhK5_10101_93 | GAAGGTGACCAAGTTCATGCTTTTTATCTTATCCTGCGATTTCCTCT | GAAGGTCGGAGTCAACGGATTTTTATCTTATCCTGCGATTTCCTCC | AGAGGAAAGTGTGGTGGAGGAAGAA |
|  | **RhK5_4359_382 (Ref)** | GAAGGTGACCAAGTTCATGCTAAGTCCGATTAGCCCTGACCGT | GAAGGTCGGAGTCAACGGATTGTCCGATTAGCCCTGACCGC | AGAAGGGCGAACAGGGCTATCAAT |
|  | **Rh_PN_SNP6K (Ref)** | GAAGGTGACCAAGTTCATGCTAGGAGATCTAGAGCCTCTTGAACA | GAAGGTCGGAGTCAACGGATTGGAGATCTAGAGCCTCTTGAACG | TGGTGGACTTGATTTTGAAGCTGAAAGAA |
| Flower: fragrance | RhMCRND_13639_80 | GAAGGTGACCAAGTTCATGCTATGTGATAGAGGAAGAGGAGCCAT | GAAGGTCGGAGTCAACGGATTGTGATAGAGGAAGAGGAGCCAC | CCGTTGGAAAATGGAGGAGAAGCAT |
|  | Rh12GR_62784_393 | GAAGGTGACCAAGTTCATGCTCTTCTTCTTCCTCAAGAGACAACTT | GAAGGTCGGAGTCAACGGATTCTTCTTCTTCCTCAAGAGACAACTC | GAGCTTTCTTCTTGACTCCGGTGTT |
|  | Rh12GR_53908_964 | GAAGGTGACCAAGTTCATGCTAAAAGGACTAGAGAAACGCGGTGT | GAAGGTCGGAGTCAACGGATTAAGGACTAGAGAAACGCGGTGC | AAAATACTTGTACGTCTTGCCAGCTGAT |
|  | RhMCRND_6741_1060 | GAAGGTGACCAAGTTCATGCTGTGCTTAGAATTTCTGGATGCACATTA | GAAGGTCGGAGTCAACGGATTGCTTAGAATTTCTGGATGCACATTG | GAATTGTTCCTTTTGTTTTCGCCAAGGAA |
|  | RhMCRND_4712_444 | GAAGGTGACCAAGTTCATGCTAAATTTTGTAAATCATTCCTTTTAATAATGCCA | GAAGGTCGGAGTCAACGGATTAAATTTTGTAAATCATTCCTTTTAATAATGCCG | CAGGCATTTTTGAAGTGCCTTCACATTTA |
|  | RhMCRND_5437_1194 | GAAGGTGACCAAGTTCATGCTGTCGACAAGTCTATTGGCATCAACT | GAAGGTCGGAGTCAACGGATTCGACAAGTCTATTGGCATCAACC | GGATCAGATTCTGAAAACGTGTGCTATTT |
|  | RhK5_18439_164 | GAAGGTGACCAAGTTCATGCTGAGCTATTGGTTGAACTGGAGAGTT | GAAGGTCGGAGTCAACGGATTAGCTATTGGTTGAACTGGAGAGTC | GGTGTGAAAACCCAAATTGAACACAACAA |
|  | **RhMCRND_11924_839 (Ref)** | GAAGGTGACCAAGTTCATGCTCAGTTTCAAATATTCAAGTTAGCAGAAATGA | GAAGGTCGGAGTCAACGGATTAGTTTCAAATATTCAAGTTAGCAGAAATGG | CCCAAATGGAAAGCTCCGTTGTGAT |
|  | RhMCRND_2744_848 | GAAGGTGACCAAGTTCATGCTCAGCAACTCTTGGTATCTTGGGT | GAAGGTCGGAGTCAACGGATTCAGCAACTCTTGGTATCTTGGGC | CAGCTCAAATGATCTGCTAGGATAAGTAA |
|  | RhK5_12307_104 | GAAGGTGACCAAGTTCATGCTTTATTTAGTGCTCAAGTATTTCTCAACT | GAAGGTCGGAGTCAACGGATTTATTTAGTGCTCAAGTATTTCTCAACC | CTTTGAACAAACCCTGTACAAAGTCGAT |
|  | RhMCRND_12686_297 | GAAGGTGACCAAGTTCATGCTCAGAAGCTGCAAAAGATTTGGTTGGT | GAAGGTCGGAGTCAACGGATTAGAAGCTGCAAAAGATTTGGTTGGC | AGTAGTCTGGTCCTAATTTTTGGCGTTTA |
|  | **Rh_FR_SNP201K (Ref)** | GAAGGTGACCAAGTTCATGCTTTCACCCAAAATTCCTTTCTTCTCGT | GAAGGTCGGAGTCAACGGATTCACCCAAAATTCCTTTCTTCTCGG | GTATAGCCCGCCGTTCCTTCTCAA |
| Petal: length | Rh12GR_92884_1039 | GAAGGTGACCAAGTTCATGCTGTAGCCTTGTTGAACCATAAGCA | GAAGGTCGGAGTCAACGGATTGTAGCCTTGTTGAACCATAAGCG | GTACTCCGGTGTTAGTCATGTATAGTAA |
|  | RhMCRND_6741_1060 | GAAGGTGACCAAGTTCATGCTGTGCTTAGAATTTCTGGATGCACATTA | GAAGGTCGGAGTCAACGGATTGCTTAGAATTTCTGGATGCACATTG | GAATTGTTCCTTTTGTTTTCGCCAAGGAA |
|  | RhK5_14720_826 | GAAGGTGACCAAGTTCATGCTACTCAGAAGCACACCGAAAACAACA | GAAGGTCGGAGTCAACGGATTCAGAAGCACACCGAAAACAACG | ACTTGGGCTGGCCTCTGGCAAA |
|  | RhK5_10683_422 | GAAGGTGACCAAGTTCATGCTGCCAAAGATTACAGACACGCTGTAT | GAAGGTCGGAGTCAACGGATTCCAAAGATTACAGACACGCTGTAC | GGAGGAAGGTGCAACATTCAATCCAA |
|  | RhK5_12478_1400 | GAAGGTGACCAAGTTCATGCTACTGCTCTAGATTCTTTGGCTGCT | GAAGGTCGGAGTCAACGGATTCTGCTCTAGATTCTTTGGCTGCC | CCAGTTTAGGATCCATCGTGGCAAT |
|  | RhK5_18872_1065 | GAAGGTGACCAAGTTCATGCTTAATGTTGAGTTTCAGTTGCATGCGT | GAAGGTCGGAGTCAACGGATTAATGTTGAGTTTCAGTTGCATGCGC | GTGTTTATTCACAAAGTCAGGGATTTCGTA |
|  | RhMCRND_982_1009 | GAAGGTGACCAAGTTCATGCTTTGAAAAGAGAAGCTTAGAACTTCTGTTA | GAAGGTCGGAGTCAACGGATTGAAAAGAGAAGCTTAGAACTTCTGTTG | ATACGAGTTTATGGTCAAGACCCTTCAA |
|  | RhK5_3530_858 | GAAGGTGACCAAGTTCATGCTATCTAAATAACTTGAAGTATTATTAGTTCCAT | GAAGGTCGGAGTCAACGGATTATCTAAATAACTTGAAGTATTATTAGTTCCAG | CCAGAGCATCAAGCTGTTATTCCACAA |
|  | RhK5_1987_433 | GAAGGTGACCAAGTTCATGCTAAGAGGTTTCAGATTGACCCAGATT | GAAGGTCGGAGTCAACGGATTAAGAGGTTTCAGATTGACCCAGATG | CCTCAAAGCCTCCACACTCCTAAAA |
|  | **Rh_PL_SNP49K (Ref)** | GAAGGTGACCAAGTTCATGCTGTATGCGCAGATGCCAGGACAT | GAAGGTCGGAGTCAACGGATTATGCGCAGATGCCAGGACAC | ATGCTTGGGTTGGTTACAGTGGGAA |

**Supplementary Table 3.** Significance of validated markers tested with Kruskal Wallis. The effect size was estimated with Eta[2] method.

| **Trait** | **Marker** | **n** | **statistic** | **df** | **p** | **method** | **effsize** | **magnitude** |
| --- | --- | --- | --- | --- | --- | --- | --- | --- |
| Young shoot: intensity of anthocyanin coloration | Rh12GR_19603_664 | 108 | 3.83 | 2 | 0.147 | Kruskal-Wallis | 0.017 | small |
|  | Rh12GR_43585_254 | 127 | 8.01 | 3 | 0.0458 | Kruskal-Wallis | 0.041 | small |
|  | RhMCRND_5216_974 | 132 | 3.76 | 4 | 0.44 | Kruskal-Wallis | -0.002 | small |
|  | RhK5_17994_176 | 144 | 7.62 | 4 | 0.107 | Kruskal-Wallis | 0.026 | small |
|  | RhK5_1892_483 | 149 | 3.45 | 2 | 0.178 | Kruskal-Wallis | 0.01 | small |
| Stem: number of prickles | RhMCRND_2230_1005 | 124 | 12.18 | 3 | 0.00678 | Kruskal-Wallis | 0.077 | moderate |
|  | Rh12GR_16070_248 | 143 | 1.33 | 2 | 0.514 | Kruskal-Wallis | -0.005 | small |
|  | RhK5_5501_1135 | 146 | 4.22 | 4 | 0.377 | Kruskal-Wallis | 0.002 | small |
|  | RhK5_4270_45 | 152 | 6.28 | 2 | 0.0433 | Kruskal-Wallis | 0.029 | small |
|  | RhMCRND_15645_551 | 152 | 3.89 | 3 | 0.274 | Kruskal-Wallis | 0.006 | small |
|  | RhMCRND_33463_124 | 153 | 5.46 | 4 | 0.244 | Kruskal-Wallis | 0.01 | small |
|  | RhK5_1809_431 | 154 | 2.33 | 4 | 0.676 | Kruskal-Wallis | -0.011 | small |
|  | RhK5_12076_566 | 155 | 0.75 | 3 | 0.86 | Kruskal-Wallis | -0.015 | small |
|  | RhMCRND_4282_2838 | 156 | 3.18 | 2 | 0.204 | Kruskal-Wallis | 0.008 | small |
|  | RhK5_432_2321 | 158 | 0.96 | 2 | 0.618 | Kruskal-Wallis | -0.007 | small |
|  | RhMCRND_12154_179 | 159 | 3.85 | 4 | 0.427 | Kruskal-Wallis | -0.001 | small |
| Leaf: intensity of green color (upper side) | Rh88_48595_614 | 148 | 1.61 | 3 | 0.658 | Kruskal-Wallis | -0.01 | small |
|  | RhK5_8654_792 | 181 | 1.3 | 3 | 0.729 | Kruskal-Wallis | -0.01 | small |
|  | RhMCRND_32494_229 | 181 | 2.14 | 3 | 0.544 | Kruskal-Wallis | -0.005 | small |
|  | RhMCRND_201_1253 | 182 | 2.83 | 2 | 0.242 | Kruskal-Wallis | 0.005 | small |
| Leaf: anthocyanin coloration | RhK5_5599_259 | 141 | 0.56929 | 2 | 0.7523 | Chi² |  |  |
|  | RhMCRND_1644_1712 | 168 | 2.6798 | 4 | 0.6127 | Chi² |  |  |
| Leaf: glossiness of upper side | RhK5_14250_324 | 175 | 49.23 | 4 | 5.22E-10 | Kruskal-Wallis | 0.266 | large |
|  | Rh12GR_4274_338 | 181 | 58.67 | 3 | 1.13E-12 | Kruskal-Wallis | 0.314 | large |
| Flower: number of petals | RhMCRND_10097_334 | 129 | 43.1 | 4 | 9.87E-09 | Kruskal-Wallis | 0.315 | large |
|  | RhMCRND_13217_328 | 142 | 35.28 | 4 | 4.08E-07 | Kruskal-Wallis | 0.228 | large |
|  | RhMCRND_760_1054 | 144 | 66.83 | 4 | 1.06E-13 | Kruskal-Wallis | 0.452 | large |
|  | Rh_PN_SNP6K | 159 | 1.71 | 3 | 0.635 | Kruskal-Wallis | -0.008 | small |
|  | RhK5_4359_382 (Ref) | 156 | 67.93 | 4 | 6.22E-14 | Kruskal-Wallis | 0.423 | large |
|  | Rh12GR_54461_324 | 163 | 60.98 | 4 | 1.81E-12 | Kruskal-Wallis | 0.361 | large |
|  | RhK5_10101_93 | 164 | 89.09 | 4 | 2.05E-18 | Kruskal-Wallis | 0.535 | large |
| Flower: fragrance | RhMCRND_2744_848 | 126 | 43.12 | 3 | 2.32E-09 | Kruskal-Wallis | 0.329 | large |
|  | Rh12GR_53908_964 | 144 | 16.94 | 4 | 0.00199 | Kruskal-Wallis | 0.093 | moderate |
|  | Rh_FR_SNP201K (Ref) | 145 | 6.4 | 4 | 0.171 | Kruskal-Wallis | 0.017 | small |
|  | RhK5_18439_164P | 150 | 42.38 | 4 | 1.39E-08 | Kruskal-Wallis | 0.265 | large |
|  | RhMCRND_12686_297 | 154 | 35.47 | 3 | 9.68E-08 | Kruskal-Wallis | 0.216 | large |
|  | RhMCRND_6741_1060 | 160 | 49.03 | 4 | 5.76E-10 | Kruskal-Wallis | 0.29 | large |
|  | Fr_RhMCRND_11924_839 (Ref) | 161 | 51.96 | 4 | 1.41E-10 | Kruskal-Wallis | 0.307 | large |
|  | Rh12GR_62784_393 | 165 | 40.92 | 4 | 2.80E-08 | Kruskal-Wallis | 0.231 | large |
|  | RhMCRND_4712_444 | 165 | 56.73 | 4 | 1.41E-11 | Kruskal-Wallis | 0.33 | large |
|  | RhK5_12307_104 | 169 | 48.17 | 4 | 8.71E-10 | Kruskal-Wallis | 0.269 | large |
|  | RhMCRND_5437_1194 | 172 | 50.1 | 4 | 3.44E-10 | Kruskal-Wallis | 0.276 | large |
| Petal: length | RhK5_12478_1400 | 123 | 5.33 | 4 | 0.255 | Kruskal-Wallis | 0.011 | small |
|  | RhMCRND_982_1009 | 131 | 23.24 | 4 | 0.000113 | Kruskal-Wallis | 0.153 | large |
|  | RhK5_3530_858 | 137 | 23.27 | 4 | 0.000112 | Kruskal-Wallis | 0.146 | large |
|  | RhMCRND_6741_1060 | 137 | 7.72 | 4 | 0.103 | Kruskal-Wallis | 0.028 | small |
|  | RhK5_1987_433 | 142 | 18.31 | 4 | 0.00107 | Kruskal-Wallis | 0.104 | moderate |
|  | RhK5_14720_826 | 144 | 1.98 | 4 | 0.739 | Kruskal-Wallis | -0.014 | small |
|  | RhK5_10683_422 | 147 | 7.92 | 4 | 0.0946 | Kruskal-Wallis | 0.028 | small |
|  | Rh_PL_SNP49K (Ref) | 150 | 10.79 | 4 | 0.029 | Kruskal-Wallis | 0.047 | small |
|  | Rh12GR_92884_1039 | 152 | 4.48 | 4 | 0.345 | Kruskal-Wallis | 0.003 | small |
|  | RhK5_18872_1065 | 153 | 19.94 | 4 | 0.000512 | Kruskal-Wallis | 0.108 | moderate |

**Supplementary Table 4.** Combinations of marker from different loci for the trait ‘Flower: fragrance’.

|  | **Rh12GR_62784_393** *η^2^*=0.231 | **Rh12GR_53908_964** *η^2^*=0.093 |
| --- | --- | --- |
| **RhMCRND_6741_1060** *η^2^*=0.29 | 0.369 | 0.274 |
| **RhMCRND_4712_444** *η^2^*=0.33 | 0.437 | 0.361 |
| **RhMCRND_5437_1194** *η^2^*=0.276 | 0.470 | 0.335 |
| **RhK5_18439_164** *η^2^*=0.265 | 0.405 | 0.341 |
| **RhMCRND_11924_839 (Ref)** *η^2^*=0.307 | 0.386 | 0.257 |
| **RhMCRND_2744_848** *η^2^*=0.329 | 0.406 | 0.387 |
| **RhK5_12307_104** *η^2^*=0.269 | 0.357 | 0.296 |
| **RhMCRND_12686_297** *η^2^*=0.216 | 0.377 | 0.268 |

**Supplementary Table 5.** Combinations of marker from different loci for the trait ‘Flower: number of petals’.

|  | **Rh12GR_54461_324** *η^2^*=0.361 | **RhMCRND_10097_334** *η^2^*=0.315 |
| --- | --- | --- |
| **RhMCRND_13217_328** *η^2^*=0.228 | 0.306 | 0.27 |
| **RhMCRND_760_1054** *η^2^*=0.452 | 0.516 | 0.521 |
| **RhK5_10101_93** *η^2^*=0.535 | 0.541 | 0.601 |
| **RhK5_4359_382** η² = 0.423 | 0.461 | 0.486 |

**Supplementary Table 6**. R² values from significant markers in the GWAS.

| **Trait** | **Marker** | **Chrom** | **Position** | **Model** | **R2** | **pval** |
| --- | --- | --- | --- | --- | --- | --- |
| Young shoot: intensity of anthocyanin content | RhK5_1892_483 | 2 | 64793994 | additive | 0.006 | 0.1995 |
|  | RhK5_1892_483 | 2 | 64793994 | 1-dom-ref | 0.003 | 0.3791 |
|  | Rh12GR_38290_1803 | 4 | 10888583 | additive | 0.092 | 0 |
|  | Rh12GR_43585_254 | 2 | 61812276 | 1-dom-alt | 0.107 | 0 |
|  | Rh12GR_30029_334 | 2 | 62046047 | 1-dom-alt | 0.099 | 0 |
|  | Rh12GR_19603_664 | 4 | 16031016 | 1-dom-alt | 0.109 | 0 |
|  | RhMCRND_5216_974 | 4 | 56389410 | 1-dom-alt | 0.095 | 0 |
|  | RhK5_17994_176 | 2 | 32185535 | 1-dom-ref | 0.083 | 0 |
| Stem: number of prickles | RhMCRND_15645_551 | 3 | 45179143 | additive | 0.082 | 0 |
|  | RhK5_12076_566 | 3 | 46342087 | additive | 0.082 | 0 |
|  | Rh12GR_16070_248 | 5 | 14391046 | 1-dom-alt | 0.081 | 0 |
|  | RhK5_1809_431 | 5 | 13294690 | 1-dom-ref | 0.085 | 0 |
| Leaf: intensity of green colour | RhMCRND_18571_180 | 6 | 47451207 | 1-dom-ref | 0.084 | 0 |
| Leaf: anthocyanin coloration | RhMCRND_1644_1712 | 4 | 54982802 | 1-dom-alt | 0.096 | 0 |
| Leaf: glossiness of upper side | Rh12GR_4274_338 | 7 | 536296 | 1-dom-ref | 0.031 | 0.004 |
|  | RhK5_14250_324 | 7 | 570928 | 1-dom-alt | 0.097 | 0 |
| Flower: number of petals | Rh12GR_54461_324 | 3 | 28842384 | additive | 0.123 | 0 |
|  | RhMCRND_10097_334 | 3 | 28989123 | additive | 0.176 | 0 |
|  | RhMCRND_13217_328 | 3 | 33149219 | additive | 0.106 | 0 |
|  | RhMCRND_760_1054 | 3 | 33215504 | additive | 0.191 | 0 |
|  | RhK5_10101_93 | 3 | 33225827 | additive | 0.193 | 0 |
|  | RhK5_4359_382 | 3 | 33559568 | additive | 0.17 | 0 |
| Flower: fragrance | RhMCRND_13639_80 | 2 | 40963137 | 1-dom-alt | 0.125 | 0 |
|  | RhMCRND_6741_1060 | 2 | 70415958 | 1-dom-alt | 0.028 | 0.0088 |
|  | RhMCRND_5437_1194 | 2 | 72294268 | additive | 0.096 | 0 |
|  | RhK5_18439_164 | 2 | 72421184 | 1-dom-ref | 0.081 | 0 |
|  | RhMCRND_11924_839 | 2 | 72493653 | 1-dom-alt | 0.088 | 0 |
|  | RhMCRND_2744_848 | 2 | 73007507 | 1-dom-alt | 0.052 | 3.00E-04 |
|  | RhK5_12307_104 | 2 | 73028954 | 1-dom-ref | 0.061 | 1.00E-04 |
|  | RhMCRND_12686_297 | 2 | 73092038 | 1-dom-alt | 0.125 | 0 |
|  | Rh12GR_53908_964 | 2 | 41894415 | 1-dom-alt | 0.118 | 0 |
|  | RhMCRND_4712_444 | 2 | 71063049 | 1-dom-alt | 0.107 | 0 |
|  | Rh12GR_62784_393 | 2 | 41519579 | 1-dom-ref | 0.108 | 0 |
| Petal: length | RhK5_14720_826 | 2 | 71149284 | additive | 0.081 | 0 |
|  | RhK5_10683_422 | 2 | 72256062 | additive | 0.096 | 0 |
